# Contextualizing the biological relevance of standardized high‐resolution respirometry to assess mitochondrial function in permeabilized human skeletal muscle

**DOI:** 10.1111/apha.13625

**Published:** 2021-03-03

**Authors:** Robert A. Jacobs, Carsten Lundby

**Affiliations:** ^1^ Department of Human Physiology & Nutrition University of Colorado Colorado Springs (UCCS) Colorado Springs CO USA; ^2^ Innland University of Applied Sciences Lillehammer Norway

**Keywords:** carbohydrate oxidation rates, fatty acid oxidation rates, human bioenergetics, metabolic flexibility, skeletal muscle mitochondria, skeletal muscle temperature

## Abstract

**Aim:**

This study sought to provide a statistically robust reference for measures of mitochondrial function from standardized high‐resolution respirometry with permeabilized human skeletal muscle (ex vivo), compare analogous values obtained via indirect calorimetry, arterial‐venous O_2_ differences and ^31^P magnetic resonance spectroscopy (in vivo) and attempt to resolve differences across complementary methodologies as necessary.

**Methods:**

Data derived from 831 study participants across research published throughout March 2009 to November 2019 were amassed to examine the biological relevance of ex vivo assessments under standard conditions, ie physiological temperatures of 37°C and respiratory chamber oxygen concentrations of ~250 to 500 μmol/L.

**Results:**

Standard ex vivo‐derived measures are lower (*Z* ≥ 3.01, *P* ≤ .0258) en masse than corresponding in vivo‐derived values. Correcting respiratory values to account for mitochondrial temperatures 10°C higher than skeletal muscle temperatures at maximal exercise (~50°C): (i) transforms data to resemble (*Z* ≤ 0.8, *P* > .9999) analogous yet context‐specific in vivo measures, eg data collected during maximal 1‐leg knee extension exercise; and (ii) supports the position that maximal skeletal muscle respiratory rates exceed (*Z* ≥ 13.2, *P* < .0001) those achieved during maximal whole‐body exercise, e.g. maximal cycling efforts.

**Conclusion:**

This study outlines and demonstrates necessary considerations when actualizing the biological relevance of human skeletal muscle respiratory control, metabolic flexibility and bioenergetics from standard ex vivo‐derived assessments using permeabilized human muscle. These findings detail how cross‐procedural comparisons of human skeletal muscle mitochondrial function may be collectively scrutinized in their relationship to human health and lifespan.

## 1 INTRODUCTION

Physical activity is integral in human health.^1^, ^2^, ^3^ Routine physical activity maintains immune function^4^ while reducing the risk of non‐communicable chronic diseases and physical disability throughout life,^3^, ^5^, ^6^ with the aggregate literature also indicating it increases life expectancy.^3^, ^7^ Alternatively, inactivity and/or reduced physical activity (ie immobility and/or bed rest), even in brief bouts,^8^, ^9^, ^10^ proceed rapid escalations in metabolic dysregulation, insulin resistance, risk of chronic disease and compromised immune function.^3^, ^4^, ^5^ Levels of physical activity,^11^ whole‐body measures of aerobic fitness,^12^ metabolic (eg ventilatory) thresholds^13^ and strength^14^ have all been identified as independent predictors of all‐cause mortality across the lifespan. A clear biological connection of these characteristics exists in their relation to skeletal muscle and, more specifically, skeletal muscle mitochondria.^15^, ^16^, ^17^, ^18^, ^19^, ^20^, ^21^

The methodological reliance on tissue‐specific respirometry has supported an exponential rise in mitochondrial research.^22^, ^23^ Arguably no other technique used to study skeletal muscle mitochondrial function has increased over the last decade more than high‐resolution respirometry (HRR) with permeabilized skeletal muscle samples. This technique eliminates free sarcoplasmic components (ie myoglobin, glycolytic enzymes, etc) by selectively perforating the sarcolemma with negligible effect on mitochondrial membranes, allowing for the isolated analysis of all skeletal muscle mitochondria in their native intracellular reticular form.^24^, ^25^, ^26^

Despite the widespread use of HRR, there is no identifiable consensus as to what characterizes human ‘*mitochondrial function*’ in relation to skeletal muscle respiratory control, metabolic flexibility or bioenergetic potential. Necessary efforts to connect the biological relevance of HRR assessments using permeabilized skeletal muscle fibres to other characteristics of human metabolism are lacking, *ie how do whole‐body rates of maximal oxygen consumption (VO_2max_) and published respiratory rates reported in pmol O_2_ per mg wet weight per second compare, and how do published respiratory rates reported in pmol O_2_ per mg wet weight per second translate to in vivo rates of substrate oxidation and/or ATP production in skeletal muscle derived from other methodologies such as indirect calorimetry (IC), arterial‐venous O_2_ differences (a‐vO_2_ diff) and ^31^P magnetic resonance spectroscopy (^31^P MRS)?* Consequently, published values representing equivalent respiratory states determined from the same skeletal muscle (*m. vastus lateralis*), using similar sample preparation techniques and comparable sample populations vary from ~27 to ~188 pmol mg^−1^ s^−1^ (~42 to ~360 mL kg^−1^ min^−1^). These values reflect approximate whole‐body VO_2max_ measures of 1.07 to 9.09 L min^−1^, which is a 10‐fold range in variability from the direct measures reported, ~30% of 3.6 L min^−1^ ^27^ to ~310% of 2.9 L min^−1^ ^28^ respectively. The latter value, 9.09 L min^−1^, is approximately 30% higher than has ever been measured in a human. In short, a comprehensive interpretation of HRR data collected using a standardized permeabilized skeletal muscle fibre technique into physiologically relevant contexts of human respiratory control, metabolism and bioenergetics is warranted.

Given the intimacy of skeletal muscle mitochondria and health, it is paramount to identify *healthy* parameters of mitochondrial *function* so that continued research efforts may differentiate and accentuate the perspective of ‘*mitochondrial dysfunction*’, as it relates to human health and ageing. Accordingly, the aims of this study are threefold: (i) Provide a statistically robust reference for measures of mitochondrial function in relation to oxygen consumption rates (OCR), substrate oxidation rates (SOR) and ATP production rates (APR) obtained using standardized HRR methodologies (ie physiological temperatures of 37°C and high respiratory chamber oxygen concentrations of ~250 to 500 μmol/L) with permeabilized human skeletal muscle samples collected from the m. vastus lateralis; (ii) Compare these ex vivo reference values to analogous measures collected with alternative in vivo methodologies (i. IC, a‐vO_2_ diff and ^31^P MRS) and (iii) Attempt to resolve differences across complementary ex vivo and in vivo methodologies as necessary. To address these aims, we amassed data across a decade of our research in combination with analogous respiratory values published across the field from 2009 to 2019 in effort to decipher the biological relevance of HRR values obtained from permeabilized human skeletal muscle samples. Collectively these findings: (i) Provide necessary reference values for respiratory measures collected using a standardized HRR methodology with permeabilized skeletal muscle samples obtained from relatively young and healthy individuals; (ii) Illustrate how these ex vivo reference values relate to analogous measures obtained using different yet valid in vivo methodologies and (iii) Identify an approach for correcting standardized HRR‐specific skeletal muscle respiratory values that improves the biological relevance and application of ex vivo‐derived indices of ‘*mitochondrial function*’.

## 2 RESULTS

### 2.1 Sample population characteristics

Data from a total of 211 internal and external sources were included for analysis and presentation; n = 159 individual measures, representing duplicate averages, were included from our own research and n = 52 obtained from published group means representing data collected from 672 individuals. External data were amassed from 23 studies published across the past decade, from March 2009 to November 2019.^29^, ^30^, ^31^, ^32^, ^33^, ^34^, ^35^, ^36^, ^37^, ^38^, ^39^, ^40^, ^41^, ^42^, ^43^, ^44^, ^45^, ^46^, ^47^, ^48^, ^49^, ^50^, ^51^ As aerobic fitness (relative whole‐body VO_2max_; mL kg^−1^ min^−1^) persists as arguably the single best predictor of all‐cause mortality to date,^12^ primary outcome variables were separated into subgroups according to aerobic fitness percentiles as specified by ACSM^52^ when controlling for age and sex. Subgroup classifications are presented hereafter as: <40th percentile (n = 10); between the 40th and 59th percentile (n = 34); between the 60th and 69th percentile (n = 45); between the 70th and 79th percentile (n = 30); between the 80th and 89th percentile (n = 36) or ≥90th percentile (n = 56). Collective group as well as individual subgroup characteristics are reported in Table 1. Main effects of aerobic fitness for age (Kruskal‐Wallis statistic = 13.7, *P* = .0177), body mass (Kruskal‐Wallis statistic = 42.2, *P* < .0001), BMI (Kruskal‐Wallis statistic = 52.6, *P* < .0001), estimated lower limb mass (Kruskal‐Wallis statistic = 26.4, *P* < .0001), absolute VO_2max_ (L min^−1^; Kruskal‐Wallis statistic = 111.7, *P* < .0001), relative VO_2max_ (mL kg^−1^ min^−1^; Kruskal‐Wallis statistic = 187.1, *P* < .0001), maximal incremental cycling power (W_max_; Kruskal‐Wallis statistic = 111.5, *P* < .0001) and relative W_max_ (W kg^−1^; Kruskal‐Wallis statistic = 186.3, *P* < .0001) were identified. Subsequent post hoc analyses detected only one difference between 40th and 59th percentile and ≥90% percentile subgroups for age (*Z* = 3.0, *P* = .0421). The only differences in body weight and leg mass were identified when compared to the ≥90% subgroup. Accordingly, there is limited evidence to suggest that minor differences in age, weight or estimated leg mass across subgroups are responsible for the subsequent findings presented. Again, all subgroup characteristic variable means, ranges and statistical comparisons are reported in Table 1.

**TABLE 1:** Total group and aerobic fitness percentile subgroup characteristics

Sub‐Group Aerobic Percentiles		Age* (y)	Weight* (kg)	BMI*	Lower Limb Mass* (kg)	VO_2max_* (L min^−1^)	VO_2max_* (mL kg^−1^ min^−1^)	W_max_* (W)	W_max_* (W kg^−1^)
(n)	(%)								
Total sample population	73.1	28.1	75.3	23.4	18.1	3.99	53.7	331.6	4.45
(n = 211)	(10‐97)	(18‐47)	(50‐120)	(18‐34)	(11.6‐24.0)	(2.2‐6.4)	(25.6‐83.5)	(176‐542)	(2.1‐7.0)
**<40%**	**24.4**	**31.4^a,b^**	**86.8^a^**	**27.1^a^**	**19.5^a,b^**	**2.80^a^**	32.3^a*^	**227.7^a^**	**2.67^a^**
(n = 10)	(9.7‐38.5)	(24‐44)	(65‐115)	(20‐34)	(16.3‐23.7)	(2.2‐3.4)	(25.6‐40.7)	(176‐280)	(2.1‐3.3)
**40%‐59%**	**51.7**	**26.2^a^**	**78.3^a^**	**24.4^a^**	**18.7^a^**	**3.37^a,b^**	**43.1^a^**	**277.3^a,b^**	**3.55^a,b^**
(n = 34)	(40.0‐59.8)	(20‐47)	(64‐93)	(20‐30)	(14.8‐20.2)	(2.8‐4.0)	(38.0‐47.7)	(227‐329)	(3.1‐3.9)
**60%‐69%**	**65.0**	**27.1^a,b^**	**78.7^a^**	**24.4^a,b^**	**18.5^a^**	**3.71^b^**	47.2^a*,b^	**306.6^b^**	**3.90^b,c^**
(n = 45)	(60.0‐69.9)	(20‐46)	(62‐93)	(19‐34)	(14.9‐20.3)	(2.9‐4.3)	(40.2‐51.2)	(239‐362)	(3.3‐4.2)
**70%‐79%**	**74.5**	**27.6^a,b^**	**73.6^a,b^**	**22.5^b,c^**	**17.9^a,b^**	**3.76^b,c^**	**51.0^b,c^**	**310.9^b,c^**	**4.22^c,d^**
(n = 30)	(70.3‐79.2)	(20‐44)	(59‐90)	(18‐26)	(15.5‐19.8)	(3.0‐4.7)	(45.1‐55.3)	(245‐393)	(3.7‐4.6)
**80%‐89%**	**83.9**	**27.6^a,b^**	**76.0^a^**	**23.3^a,b^**	**18.1^a^**	**4.28^c,d^**	**56.2^c^**	**356.8^c,d^**	**4.68^d^**
(n = 36)	(80.0‐88.7)	(19‐40)	(53‐120)	(21‐30)	(12.7‐24.0)	(2.8‐6.4)	(46.4‐65.4)	(227‐542)	(3.8‐5.5)
**≥90%**	**93.8**	**30.2^b^**	**69.2^b^**	**21.7^c^**	**17.2^b^**	**4.76^d^**	**68.8^d^**	**398.1^d^**	**5.75^e^**
(n = 56)	(90.0‐97.0)	(18‐45)	(50‐90)	(19‐26)	(11.6‐20.0)	(2.5‐6.0)	(50.6‐83.5)	(204‐507)	(4.1‐7.0)

Note

Means are shown in bold over minimum‐maximum values in parentheses. Characteristics across subgroups were analysed using a non‐parametric ANOVA (Kruskal‐Wallis) test and main effects evaluated with Dunn's multiple‐comparison test to control type I error. Different superscripted letters represent significant differences across subgroups (*P* < .05) and *indicates *Z* = 2.845, *P* = .0666. Maximal rates of whole‐body oxygen consumption (VO_2max_) and maximal power output (W_max_), estimated as the Watt value calculated from the following formula: VO_2max_ = 0.16 + (0.0117 × W_max_).^142^

Bold represents the mean for values in the table beginning from 73.1 in the upper left portion of the table and ending in 5.75 in the lower right portion of the table.

#### 2.1.1 Maximal human skeletal muscle fatty acid oxidation rates (FAO*_p_*)

**FIGURE 1:**
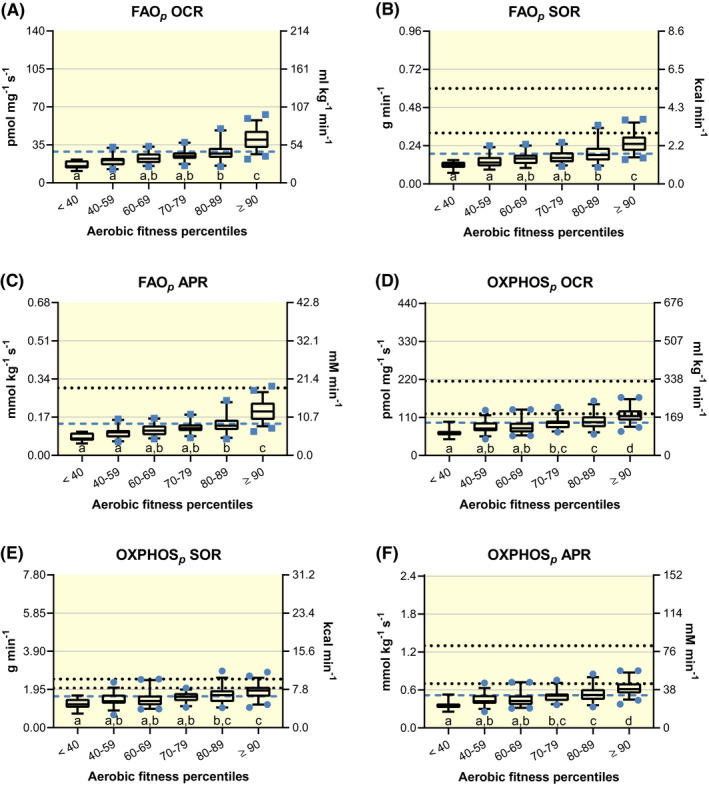
Standardized high‐resolution respirometry‐derived rates of oxygen consumption (OCR), substrate oxidation (SOR) and ATP production (APR) with permeabilized human skeletal muscle. Box and 95% confidence interval‐whisker plots across aerobic fitness percentile subgroups with a blue‐dashed line identifying total group mean. Different letters represent significant differences across subgroups (*P* < .05). Well‐coupled respiration (P) representative of mitochondrial fatty acid oxidation rates (FAO*_p_*) is represented by blue squares (n = 189), A‐C; P‐state rates of mitochondrial oxidative phosphorylation (OXPHOS*_p_*) are represented by blue circles (n = 211), D‐F. Respiratory states and aerobic fitness were analysed using one‐way analysis of variance (ANOVA) assuming Gaussian distribution of residuals. A non‐parametric one‐way ANOVA (Kruskal‐Wallis test) was instead used once this assumption was violated. Significant main effects were evaluated using Bonferroni's or Dunn's multiple‐comparison test respectively, to control type I error. For reference: In vivo measures of maximal whole‐body fat oxidation rates (MFO) in untrained controls (0.32 g min^−1^; lower dotted line) and endurance athletes (0.60 g min^−1^; upper dotted line),^53^ B; a long‐standing reference^57^, ^58^ of maximal APR derived from FAO, 0.30 mmol kg^−1^ s^−1^ is indicated by dotted line, C; average in vivo OCR obtained via arteriovenous oxygen differences during maximal two‐legged cycling efforts (184.7 mL kg^−1^ min^−1^; lower dotted line)^36^, ^67^ and one‐legged kicking (328.9 mL kg^−1^ min^−1^; upper dotted line),^28^, ^75^ D; one‐leg estimates of carbohydrate (CHO)‐specific respiration at maximal cycling efforts from moderately active individuals (2.02 g min^−1^; lower dotted line) and professional endurance athletes (2.48 g min^−1^; upper dotted line),^84^ E; and long‐standing estimates of maximal APR derived from CHO‐specific respiration (lower dotted line) and glycolysis (upper dotted line) of 0.70 and 1.3 mmol kg^−1^ s^−1^ respectively,^57^, ^58^ F

Maximal state 3 rates of well‐coupled respiration (P) with lipid substrates (octanoyl‐ or‐ palmitoyl‐carnitine) supplying maximal electron input to the Q‐cycle from the electron‐transferring flavoprotein complex with some simultaneous malate‐driven electron input via NADH dehydrogenase are experimentally administered to represent maximal rates of mitochondrial fatty acid oxidation (FAO*_p_*) in skeletal muscle. Descriptive statistics for the portion of the collective group (n = 211) that reported FAO*_p_* (n = 189) are shown in Table 2 and subgroup data separated by aerobic fitness percentiles are displayed in Figure 1A‐C. There is a main effect of aerobic fitness on OCR (Kruskal‐Wallis statistic ≥106.6, *P* < .0001), SOR (Kruskal‐Wallis statistic ≥87.9, *P* < .0001) and APR (Kruskal‐Wallis statistic ≥106.5, *P* < .0001). The group mean as well as fitness‐matched measures of fat oxidation (g min^−1^) fall below respective measures of IC‐derived maximal rates of whole‐body fat oxidation (MFO).^53^, ^54^, ^55^, ^56^ Additionally, all but one estimated APR are lower than the purported maximal rate of ATP production derived from FAO, 0.30 mmol kg^−1^ s^−1^.^57^, ^58^ Standardized (37°C and high chamber oxygen concentrations) HRR‐derived measures of FAO*_p_* from permeabilized skeletal muscle samples appear relatively lower than related literature examining analogous in vivo measures of human skeletal muscle fat metabolism, such as with IC methodologies.

**TABLE 2:** Total group descriptive statistics for standardized high‐resolution respirometry‐derived maximal rates of mitochondrial fatty acid oxidation (FAO*_p_*) from permeabilized human skeletal muscle samples

FAO*_p_*	OCR	OCR	SOR	SOR	APR	APR
n = 189	pmol mg^−1^ s^−1^	mL kg^−1^ min^−1^	g min^−1^	kcal min^−1^	mmol kg^−1^ s^−1^	mmol/L min^−1^
Minimum	10.9	16.6	0.07	0.60	0.053	3.3
25% Percentile	21.3	32.6	0.14	1.26	0.104	6.6
Median	26.5	40.5	0.18	1.61	0.130	8.2
75% Percentile	34.4	52.8	0.24	2.11	0.169	10.6
Maximum	62.8	96.3	0.41	3.66	0.308	19.4
Range	51.9	79.7	0.34	3.06	0.255	16.1
Mean	28.8	44.1	0.19	1.73	0.141	8.9
Std. Deviation	10.7	16.5	0.07	0.62	0.053	3.3
Lower 95% CI of mean	27.3	41.8	0.18	1.64	0.134	8.4
Upper 95% CI of mean	30.4	46.5	0.20	1.82	0.149	9.4
Coefficient of variation	37.3%	37.3%	36.3%	36.1%	37.3%	37.3%

Abbreviations: APR, ATP production rates; OCR, oxygen consumption rates; SOR, substrate oxidation rates.

#### 2.1.2 Maximal human skeletal muscle oxidative phosphorylation rates (OXPHOS*_p_*)

**FIGURE 2:**
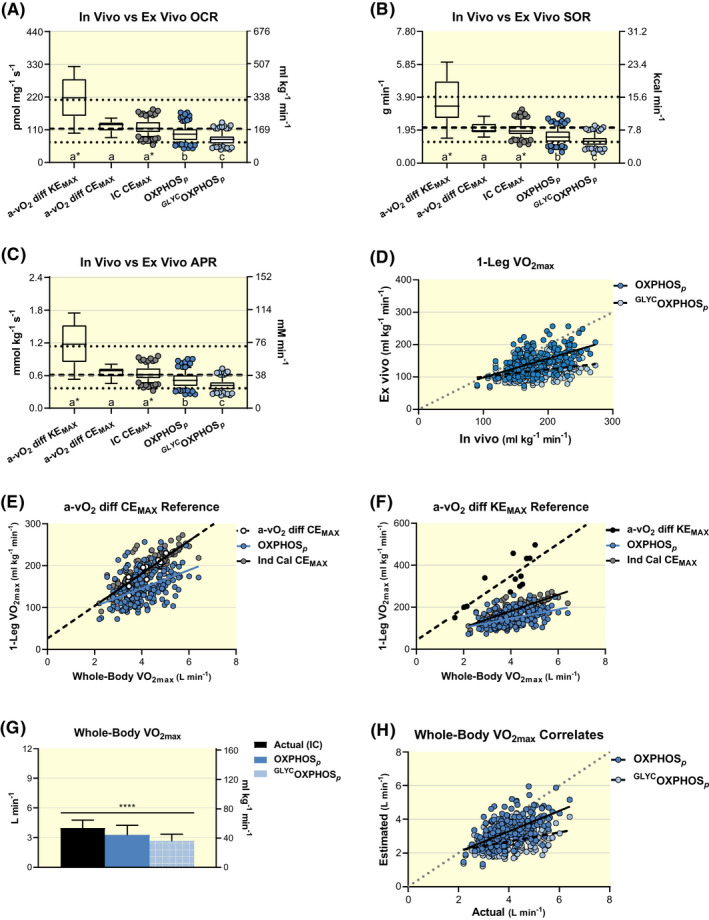
Evaluations of analogous values derived using standardized high‐resolution respirometry (HRR) with permeabilized human skeletal muscle (ex vivo, n = 211) when compared to indirect calorimetry (IC, n = 211), arteriovenous oxygen difference (a‐vO_2_ diff) during maximal knee extension (KE_MAX_, n = 13) and whole‐body cycling exercise (CE_MAX_, n = 11) and ^31^P magnetic resonance spectroscopy (^31^P MRS, n = 32) methodologies (in vivo). Ex vivo respiratory states representing well‐coupled (P) rates of oxidative phosphorylation (OXPHOS*_p_*) as well as OXPHOS*_p_* considering the repressive influence of glycolytic energetics on cellular respiration (^GLYC^OXPHOS*_p_*)^76^, ^77^ are presented. Box and 95% confidence interval‐whisker plots across methodologies comparing oxygen consumption rates (OCR), A; substrate oxidation rates (SOR), B; ATP production rates (APR) and C, with lower dotted, middle dashed and upper dotted lines representing minimum, mean and maximum ^31^P MRS‐derived values from quadricep muscle during exercise across 32 studies respectively, previously reviewed (*data extracted from figure 9D in reference*).^80^ Different letters represent significant differences across methodologies (*P* < .05) and *indicates 0.0591 ≤ *P* ≤ .0992 across respective methodologies. Representative measures of respiratory control, metabolic flexibility and energetics across methodologies were analysed using a non‐parametric ANOVA (Kruskal‐Wallis test) and main effects evaluated with Dunn's multiple‐comparison test to control type I error. Paired ex vivo to in vivo (IC) estimates of maximal rates of oxygen consumption (VO_2max_) for one leg at CE_MAX_, D; relationships between whole‐body and one‐leg VO_2max_ correlates estimated from HRR‐ and‐ IC‐derived values compared to direct a‐vO_2_ diff assessments during CE_MAX_ and KE_MAX_ in E and F respectively; and paired ex vivo‐derived estimates relative to direct in vivo (IC) assessments of whole‐body VO_2max_, H. Simple linear regression analyses were used to evaluate relationships and comparisons between respective regression lines were evaluated as significant at *P* < .01 to control for type 1 error. Actual IC‐assessed measures of whole‐body VO_2max_ were compared to OXPHOS*_p_*‐ and ^GLYC^OXPHOS*_p_*‐derived estimates with repeated measures ANOVA and post hoc pair‐wise evaluations with Bonferroni's multiple‐comparison test to control type I error, G

Well‐coupled P‐state respiration with maximal convergent flow of electrons into the Q‐cycle from NADH dehydrogenase via malate, pyruvate and/or glutamate as well as succinate dehydrogenase via succinate are experimentally administered to represent maximal rates of mitochondrial oxidative phosphorylation (OXPHOS*_p_*) in skeletal muscle. Descriptive statistics (n = 211) are reported in Table 3 and subgroup data separated by aerobic fitness percentiles are displayed in Figure 1D‐F. There is a main effect of aerobic fitness on OCR (*F* ≥ 21.5, *P* < .0001), SOR (*F* ≥ 10.4, *P* < .0001) and APR (*F* ≥ 21.5, *P* < .0001). There are also main effects of methodology used to calculate OCR (Kruskal‐Wallis statistic ≥271.1, *P* < .0001), SOR (Kruskal‐Wallis statistic ≥266.4, *P* < .0001) and APR (Kruskal‐Wallis statistic ≥272.6, *P* < .0001) when comparing ex vivo HRR‐derived values to in vivo paired IC and complementary a‐vO_2_ diff‐derived measures (Figure 2A‐C). Values relating to a‐vO_2_ diff were determined during maximal normoxic 2‐leg cycling exercise (CE_MAX_; n = 11 group averages), as reported across 10 different studies^36^, ^67^ or maximal 1‐leg knee extension efforts (KE_MAX_; n = 13 group averages), as reported across 11 different studies.^28^, ^75^ HRR‐derived measures of OCR (*Z* ≥ 3.01, *P* ≤.0258), SOR (*Z* ≥ 3.19, *P* ≤.0144) and APR (*Z* = 3.02, *P* ≤ .0255) are all lower than corresponding in vivo‐derived estimates (IC and a‐vO_2_ diff). All comparisons are worse when accounting for the repressive influence of glycolytic ATP production on cellular respiration^76^ (^GLYC^OXPHOS*_p_*). Glycolytically derived ATP alters the cellular adenylate equilibrium by increasing the ratio of ATP to ADP + inorganic phosphate (P_i_) and subsequent free energy associated with ATP hydrolysis (ΔG_ATP_), which results in more back pressure on ATP synthase and reduces the rate of ATP production.^77^ Previous studies comparing in vivo and ex vivo skeletal muscle OCR have not considered glycolytic repression of skeletal muscle respiration.^28^, ^65^, ^78^, ^79^ It is important to note: (i) IC‐derived estimates of maximal 1‐leg OCR (*Z* ≤ 0.37, *P* > .9999), SOR (*Z* ≤ 0.59, *P* > .9999) and APR (*Z* = 0.36, *P* > .9999) are not different from a‐vO_2_ diff at CE_MAX_ and they appear to correspond well to ^31^P MRS‐derived estimates^80^ (Figure 2A‐C); and (ii) Measures of whole‐body VO_2max_ in this study (Table 1) are comparable (*F* = 0.77, *P* = .4634) to reported values in studies utilizing a‐vO_2_ diff to determine OCR during CE_MAX_ ^36^, ^67^ and KE_MAX_ ^28^, ^75^ (3.99 vs 4.12 vs 3.75 L min^−1^ respectively).

**TABLE 3:** Total group descriptive statistics for standardized high‐resolution respirometry‐derived maximal rates of well‐coupled (P) oxidation phosphorylation (OXPHOS*_p_*) from permeabilized human skeletal muscle samples

OXPHOS*_p_*	OCR	OCR	SOR	SOR	APR	APR
n = 211	pmol mg^−1^ s^−1^	mL kg^−1^ min^−1^	g min^−1^	kcal min^−1^	mmol kg^−1^ s^−1^	mmol/L min^−1^
Minimum	47.1	72.2	0.65	2.62	0.256	16.1
25% Percentile	74.3	114.1	1.26	5.03	0.403	25.4
Median	94.3	144.8	1.55	6.19	0.512	32.2
75% Percentile	112.3	172.4	1.88	7.54	0.610	38.4
Maximum	166.9	256.6	2.89	11.58	0.906	57.0
Range	119.8	184.4	2.24	8.96	0.650	40.9
Mean	94.9	146.0	1.60	6.38	0.516	32.5
Std. Deviation	24.7	38.2	0.42	1.66	0.134	8.5
Lower 95% CI of mean	91.6	140.8	1.54	6.16	0.497	31.3
Upper 95% CI of mean	98.3	151.2	1.65	6.61	0.534	33.6
Coefficient of Variation	26.0%	26.1%	26.0%	26.0%	26.0%	26.1%

Abbreviations: APR, ATP production rates; OCR, oxygen consumption rates; SOR, substrate oxidation rates.

The slope of paired in vivo (IC) and ex vivo (HRR) correlates differ significantly (*F* = 42.6, *R* ^2^ = 0.29, *P* < .0001) from a perfect relationship (*r* = 1.0; Figure 2D). The discrepancy between in vivo and ex vivo paired correlates grow worse (*F* = 231.1, *P* < .0001) when accounting for the repressive influence of glycolytic ATP production on cellular respiration at CE_MAX_. Slopes of maximal 1‐leg OCR relative to whole‐body VO_2max_ (L min^−1^) for a‐vO_2_ diff‐derived values at CE_MAX_ and OXPHOS*_p_* do not differ (*F* = 1.51, *P* = .2201) but the y‐intercept for OXPHOS*_p_* is higher (*F* = 11.5, *P* = .0008; Figure 2E). Slopes of maximal 1‐leg OCR relative to whole‐body VO_2max_ for OXPHOS*_p_* and ^GLYC^OXPHOS*_p_* differ from a‐vO_2_ diff‐derived values during KE_MAX_ (Figure 2F) and both KE_MAX_ and CE_MAX_, respectively (*F* ≥ 6.9, *P* ≤ .0091; ^GLYC^OXPHOS*_p_* correlates not shown). Again, it is important to note that the correlative relationships of maximal 1‐leg OCR relative to whole‐body VO_2max_ (L min^−1^) are not different (slope *F* = 0.22, *P* = .6421; y‐intercept *F* = 0.18, *P* = .6749) between IC‐derived and a‐vO_2_ diff when assessed at CE_MAX_ (Figure 2E). Alternatively, that same relationship is different (*F* = 35.6, *P* < .0001) when comparing IC‐derived values at CE_MAX_ and a‐vO_2_ diff at KE_MAX_ (Figure 2F). Collectively, observations reported in Figure 2A,E,F (ie the similarities between IC vs a‐vO_2_ diff during CE_MAX_ but not during KE_MAX_) support our calculations of maximal 1‐leg OCRs from IC‐derived measures of VO_2max_ (L min^−1^).

There is a main effect of methodology to determine whole‐body VO_2max_ (L min^−1^ and mL kg^−1^ min^−1^; *F* ≥ 380.7, *P* < .0001), as extrapolated OXPHOS*_p_* (*t* ≥ 14.7, *P* < .0001) and ^GLYC^OXPHOS*_p_* (*t* ≥ 27.6, *P* < .0001) are lower than actual IC‐derived measures of whole‐body VO_2max_ (Figure 2G). The slope of paired IC‐ and HRR‐derived VO_2max_ correlates differ significantly (*F* = 39.2, *P* < .0001) from a perfect relationship, which becomes worse (*F* = 200.2, *P* < .0001) when accounting for the repressive influence of glycolytic ATP production on cellular respiration (Figure 2H).

Collectively, standardized HRR‐derived measures reflecting OXPHOS*_p_* from permeabilized human skeletal muscle samples are comparatively lower than analogous in vivo measures derived from IC and a‐vO_2_ diff methodologies, and also appear lower than values obtained with ^31^P MRS (see dotted and dashed lines in Figure 2A‐C).

#### 2.1.3 Maximal human skeletal muscle electron transport system rates (ETS)

Maximal rates of non‐coupled respiration (E) with analogous electron flow into the Q‐cycle as OXPHOS*_p_* are commonly referred to as the electron transfer state (ETS) and discussed as the respiratory state that is uninhibited by phosphorylative restraint. Descriptive statistics (n = 187) are reported in Table 4 and group data separated by aerobic fitness percentiles are displayed in Figure S1. There is a main effect of aerobic fitness on OCRs (Kruskal‐Wallis statistic ≥71.3, *P* < .0001). As this respiratory state represents non‐coupled respiration, APR are not applicable to these measures and comparative physiological measures of SOR for this respiratory state are not known. Thus, SOR and APR are not calculated or reported.

**TABLE 4:** Total group descriptive statistics for standardized high‐resolution respirometry‐derived maximal rates of non‐coupled respiration representative of electron transport system capacity (ETS) from permeabilized human skeletal muscle samples

ETS	OCR	OCR
n = 187	pmol mg^−1^ s^−1^	mL kg^−1^ min^−1^
Minimum	52.1	80.1
25% Percentile	89.2	136.8
Median	112.7	173.0
75% Percentile	135.1	207.5
Maximum	202.5	311.4
Range	150.4	231.3
Mean	114.9	176.7
Std. Deviation	31.5	48.6
Lower 95% CI of mean	110.4	169.7
Upper 95% CI of mean	119.4	183.7
Coefficient of Variation	27.4%	27.5%

Abbreviation: OCR, oxygen consumption rates.

### 2.2 Temperature‐corrected respiratory rates

The discrepancy between corresponding in vivo (IC) measures collected during CE_MAX_ and complimentary ex vivo (HRR) paired correlates widen with increasing OCR, as ex vivo‐in vivo differences become progressively more negative (Figure 2D,E,H). Initially, chamber oxygen concentration was considered as possibly limiting when analysing skeletal muscle samples from more fit individuals even though data included in this study utilized high chamber oxygen concentrations (250‐500 μmol/L), to the best of our knowledge. While there is a slightly negative yet significant relationship between chamber oxygen concentration and aerobic fitness‐normalized measures of OXPHOS*_p_* (Figure S2; *R* ^2^ = 0.0435, *F* = 4.04, *P* = .0474), as identified from a subset (DFn, DFd = 1, 88) of our data that were immediately available, chamber oxygen concentration cannot alone explain the discrepancy between complimentary in vivo and ex vivo measures of OCR, SOR or APR. Next, the role of temperature on respiratory rates was considered to explain the divergence between like in vivo and ex vivo measures. While respiratory chamber temperature has been largely standardized for research at or around physiological temperatures of 37°C,^25^, ^81^ respiring mitochondria have been reported to function at temperatures reaching over 50°C or ~10°C higher than the enveloping cell.^82^, ^83^ Accordingly, we adjusted all respiratory measures to control for ostensibly lower artificial temperatures during standardized data collection as detailed in the methods.

#### 2.2.1 Temperature‐corrected FAO*_p_* (^TEMP^FAO*_p_*)

**FIGURE 3:**
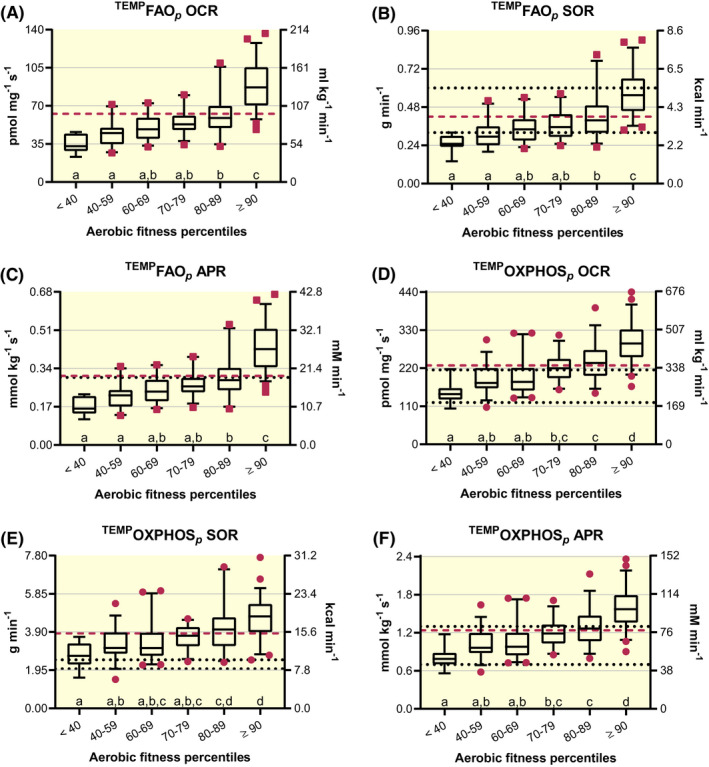
Temperature‐corrected high‐resolution respirometry‐derived rates of oxygen consumption (OCR), substrate oxidation (SOR) and ATP production (APR) with permeabilized human skeletal muscle. Box and 95% confidence interval‐whisker plots across aerobic fitness percentile subgroups with a red‐dashed line identifying total group mean. Different letters represent significant differences across subgroups (*P* < .05). Well‐coupled respiration (P) representative of mitochondrial fatty acid oxidation rates (FAO*_p_*) is represented by red squares (n = 189), A‐C; and P‐state rates of mitochondrial oxidative phosphorylation (OXPHOS*_p_*) are represented by red circles (n = 211) in D‐F. Respiratory states and aerobic fitness were analysed using one‐way analysis of variance (ANOVA) assuming Gaussian distribution of residuals. A non‐parametric one‐way ANOVA (Kruskal‐Wallis test) was instead used once this assumption was violated. Significant main effects were evaluated using Bonferroni's or Dunn's multiple‐comparison test respectively, to control type I error. For reference: In vivo measures of maximal whole‐body fat oxidation rates (MFO) in untrained controls (0.32 g min^−1^; lower dotted line) and endurance athletes (0.60 g min^−1^; upper dotted line),^53^ B; a long‐standing reference^57^, ^58^ of maximal APR derived from FAO, 0.30 mmol kg^−1^ s^−1^ is indicated by dotted line, C; average in vivo OCR obtained via arteriovenous oxygen differences during maximal two‐legged cycling efforts (184.7 mL kg^−1^ min^−1^; lower dotted line)^36^, ^67^ and one‐legged kicking (328.9 mL kg^−1^ min^−1^; upper dotted line),^28^, ^75^ D; one‐leg estimates of carbohydrate (CHO)‐specific respiration at maximal cycling efforts from moderately active individuals (2.02 g min^−1^; lower dotted line) and professional endurance athletes (2.48 g min^−1^; upper dotted line),^84^ E and long‐standing estimates of maximal APR derived from CHO‐specific respiration (lower dotted line) and glycolysis (upper dotted line) of 0.70 and 1.3 mmol kg^−1^ s^−1^ respectively,^57^, ^58^ F

Temperature correcting FAO*_p_* was determined as 45% of maximal corrections with mean femoral venous temperature estimates of 38.2°C, ranging from 37.9 to 38.7°C, to approximate more appropriate skeletal muscle and mitochondrial temperatures at an exercise intensity (percentage of VO_2max_) in which maximal rates of fat oxidation (FAT_MAX_) are commonly reported.^53^, ^54^, ^55^ Descriptive statistics are reported in Table S1 and subgroup data separated by aerobic fitness percentiles are displayed in Figure 3A‐C. There is a main effect of aerobic fitness on OCR (Kruskal‐Wallis statistic ≥108.7, *P* < .0001), SOR (Kruskal‐Wallis statistic ≥89.7, *P* < .0001) and APR (Kruskal‐Wallis statistic ≥108.7, *P* < .0001). Now, ^TEMP^FAO*_p_* (g min^−1^) appears comparable to representative and fitness‐matched measures of MFO obtained with IC^53^, ^54^, ^55^, ^56^ (Figure 3B). Specifically, a group of untrained young men (n = 8, 24 years and VO_2max_ of 48 mL kg^−1^ min^−1^) and endurance‐trained male cross‐country skiers (n = 8, 21 years and VO_2max_ of 71 mL kg^−1^ min^−1^) presented with average MFO rates of 0.32 and 0.60 g min^−1^ respectively.^53^ Those rates compare favourably to respective aerobic fitness‐matched ^TEMP^FAO*_p_* of 0.34 g min^−1^ (60‐69th percentile) and 0.57 g min^−1^ (≥90th percentile; Figure 3B). Additionally, the collective ^TEMP^FAO*_p_*‐specific group APR mean of 0.307 mmol kg^−1^ s^−1^ (Figure 3C and Table S1) closely resembles traditionally espoused rates of fat‐driven ATP synthesis (0.30 mmol kg^−1^ s^−1^; Figure 3C).^57^, ^58^ Correcting for temperature across standardized HRR‐derived FAO*_p_* appears to adjust respiratory values so that they compare favourably with related literature examining equivalent measures of human skeletal muscle fat metabolism using in vivo methodologies such as IC.

#### 2.2.2 Temperature‐corrected OXPHOS*_p_* (^TEMP^OXPHOS*_p_*)

Descriptive statistics are reported in Table S2 and subgroup data separated by aerobic fitness percentiles are displayed in Figure 3D‐F. There is a main effect of aerobic fitness on ^TEMP^OCR (*F* ≥ 30.7, *P* < .0001), ^TEMP^SOR (Kruskal‐Wallis statistic ≥59.7, *P* < .0001) and ^TEMP^APR (*F* = 30.7, *P* < .0001). Estimated rates of ^TEMP^SOR appear higher than IC‐derived estimated rates of carbohydrate (CHO) oxidation during CE_MAX_ ^84^ (Figure 3E). Estimated ^TEMP^APR (1.24 mmol kg^−1^ s^−1^) also appear higher than traditionally espoused rates of aerobic CHO‐driven ATP synthesis (0.70 mmol kg^−1^ s^−1^) but approach reported rates of glycolytic ATP synthesis (1.30 mmol kg^−1^ s^−1^)^57^, ^58^ (Figure 3F).

**FIGURE 4:**
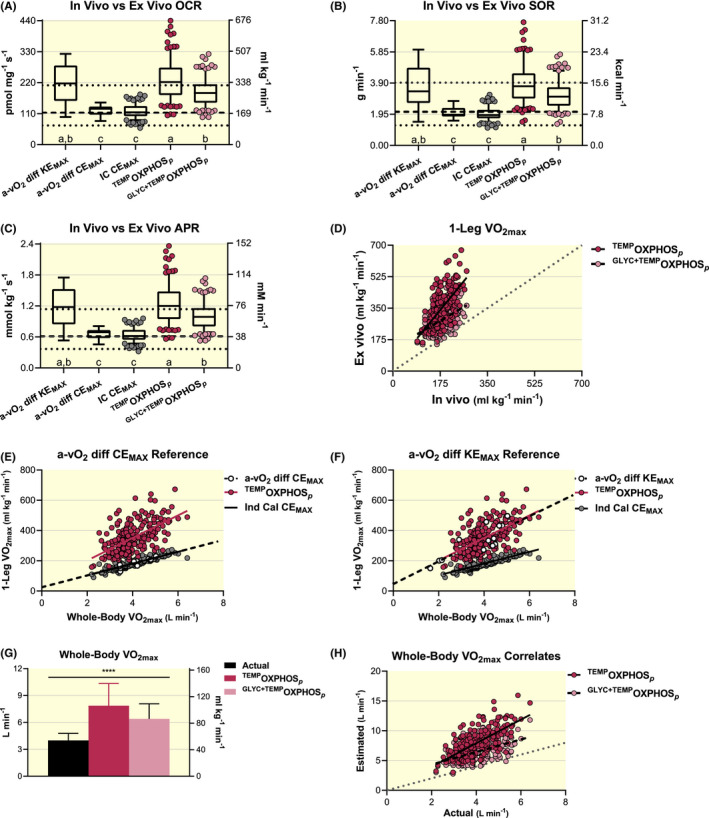
Evaluations of analogous values derived using temperature‐controlled high‐resolution respirometry (HRR) with permeabilized human skeletal muscle (ex vivo, n = 211) when compared to indirect calorimetry (IC, n = 211), arteriovenous oxygen difference (a‐vO_2_ diff) during maximal knee extension (KE_MAX_, n = 13) and whole‐body cycling exercise (CE_MAX_, n = 11) and ^31^P magnetic resonance spectroscopy (^31^P MRS, n = 32) methodologies (in vivo). Temperature‐controlled ex vivo respiratory states representing well‐coupled (P) rates of oxidative phosphorylation (^TEMP^OXPHOS*_p_*) as well as ^TEMP^OXPHOS*_p_* considering the repressive influence of glycolytic energetics on cellular respiration (^GLYC+TEMP^OXPHOS*_p_*)^76^, ^77^ are presented. Box and 95% confidence interval‐whisker plots across methodologies comparing oxygen consumption rates (OCR), A; substrate oxidation rates (SOR), B; and ATP production rates (APR), C; with lower dotted, middle dashed and upper dotted lines representing minimum, mean and maximum ^31^P MRS‐derived values from quadricep muscle during exercise across 32 studies respectively, previously reviewed (*data extracted from figure 9D in reference*).^80^ Different letters represent significant differences across methodologies (*P* < .05). Representative measures of respiratory control, metabolic flexibility and energetics across methodologies were analysed using a non‐parametric ANOVA (Kruskal‐Wallis test) and main effects evaluated with Dunn's multiple‐comparison test to control type I error. Paired ex vivo to in vivo (IC) estimates of maximal rates of oxygen consumption (VO_2max_) for one leg at CE_MAX_, D; relationships between whole‐body and one‐leg VO_2max_ correlates estimated from HRR‐ and IC‐derived values compared to direct a‐vO_2_ diff assessments during CE_MAX_ and KE_MAX_ in E and F respectively; and paired ex vivo‐derived estimates relative to direct in vivo (IC) assessments of whole‐body VO_2max_, H. Simple linear regression analyses were used to evaluate relationships and comparisons between respective regression lines were evaluated as significant at *P* < .01 to control for type 1 error. Actual IC‐assessed measures of whole‐body VO_2max_ were compared to ^TEMP^OXPHOS*_p_*‐ and ^GLYC+TEMP^OXPHOS*_p_*‐derived estimates with repeated measures ANOVA and post hoc pair‐wise evaluations with Bonferroni's multiple‐comparison test to control type I error, G

There are main effects of methodology used to calculate OCR (Kruskal‐Wallis statistic ≥370.9, *P* < .0001), SOR (Kruskal‐Wallis statistic ≥365.2, *P* < .0001) and APR (Kruskal‐Wallis statistic ≥272.6, *P* < .0001) when comparing ex vivo ^TEMP^HRR‐derived values to in vivo paired IC and complementary a‐vO_2_ diff‐derived measures (Figure 4A‐C). HRR‐derived measures of ^TEMP^OCR (*Z* ≥ 5.63, *P* < .0001), ^TEMP^SOR (*Z* ≥ 5.34, *P* < .0001) and ^TEMP^APR (*Z* = 5.65, *P* < .0001) are all higher than paired and corresponding in vivo IC and a‐vO_2_ diff at CE_MAX_ respectively. However, ^TEMP^OXPHOS*_p_* OCR (*Z* = 0.75, *P* > .9999), SOR (*Z* = 0.65, *P* > .9999) and APR (*Z* = 0.75, *P* > .9999) do not differ from values determined with a‐vO_2_ diff at KE_MAX_. Thus, ^TEMP^OXPHOS*_p_* OCR are now comparable to a‐vO_2_ diff during KE_MAX_ (350.4 vs 328.9 mL kg^−1^ min^−1^ respectively) but higher than a‐vO_2_ diff at CE_MAX_ (184.7 mL kg^−1^ min^−1^; Figures 3D and 4A). Accounting for glycolysis lowers ^GLYC+TEMP^OCR (*Z* ≥ 5.11, *P* < .0001), ^GLYC+TEMP^SOR (*Z* ≥ 5.03, *P* < .0001) and ^GLYC+TEMP^APR (*Z* ≥ 5.12, *P* < .0001) from ^TEMP^OXPHOS*_p_*‐derived values but overall comparisons across ex vivo and in vivo methodologies are the same regardless of glycolytic consideration (Figure 4A‐C).

Paired in vivo (IC) and ex vivo (^TEMP^OXPHOS*_p_*) correlates differ significantly from a perfect relationship (*F* = 30.3, *P* < .0001) but now in the opposite direction, as ex vivo‐in vivo differences become progressively more positive with increasing OCR (Figure 4D). The slope of paired IC and ^GLYC+TEMP^OXPHOS*_p_* correlates do not differ (*F* = 1.077, *P* = .3000) yet the y‐intercept for ^GLYC+TEMP^OXPHOS*_p_* is ~125% higher (*F* = 606.4, *P* < .0001; Figure 4D). Slopes of maximal 1‐leg OCR (mL kg^−1^ min^−1^) relative to whole‐body VO_2max_ (L min^−1^) for both ^TEMP^OXPHOS*_p_* (*F* = 0.59, *P* = .4449; Figure 4E) and ^GLYC+TEMP^OXPHOS*_p_* (*F* = 0.05, *P* = .8253; data not shown) are similar to a‐vO_2_ diff at CE_MAX_, although they both have higher y‐intercepts (*F* = 50.9 and 31.3 respectively; both, *P* < .0001). Alternatively, the corresponding relationships of maximal 1‐leg OCRs relative to whole‐body VO_2max_ are the same between ^TEMP^OXPHOS*_p_* and a‐vO_2_ diff during KE_MAX_ (slopes: *F* < 0.01, *P* = .9471; and y‐intercepts: *F* = 0.02, *P* = .8858; Figure 4F). Slopes between ^GLYC+TEMP^OXPHOS*_p_* and a‐vO_2_ diff during KE_MAX_ are also considered statistically similar to the α′‐level (0.01) adjusted to control for type I error across multiple comparisons (5),^85^ as described in the Methods (*F* = 5.88, *P* = .0161; data not shown).

There is a main effect for methodology to determine whole‐body VO_2max_ (L min^−1^ and mL kg^−1^ min^−1^; *F* ≥ 727.1, *P* < .0001), as extrapolated ^TEMP^OXPHOS*_p_* (*t* ≥ 37.7, *P* < .0001) and ^GLYC+TEMP^OXPHOS*_p_* (*t* ≥ 23.4, *P* < .0001) are higher than actual IC‐derived measures of whole‐body VO_2max_ (Figure 4G). The slope of paired ^TEMP^OXPHOS*_p_* and IC‐derived VO_2max_ correlates differ significantly (*F* = 42.6, *P* < .0001) from a perfect relationship (*r* = 1.0). The slope of paired IC and ^GLYC+TEMP^OXPHOS*_p_* correlates do not differ (*F* = 0.18, *P* = .6699), yet the y‐intercept for ^GLYC+TEMP^OXPHOS*_p_* is higher (*F* = 633.1, *P* < .0001; Figure 4H).

Collectively, it appears that temperature‐corrected HRR‐derived measures from permeabilized human skeletal muscle samples resemble in vivo measures obtained during KE_MAX_ but are higher than complementary in vivo assessments collected during maximal CE_MAX_.

#### 2.2.3 Temperature‐corrected ETS (^TEMP^ETS)

Descriptive statistics are reported in Table S3 and group data separated by aerobic fitness percentiles are displayed in Figure S3. There is a main effect of aerobic fitness on OCRs (Kruskal‐Wallis statistic ≥84.8, *P* < .0001).

### 2.3 Excess aerobic energetic potential of skeletal muscle when compared to values achieved at maximal whole‐body exercise efforts

**FIGURE 5:**
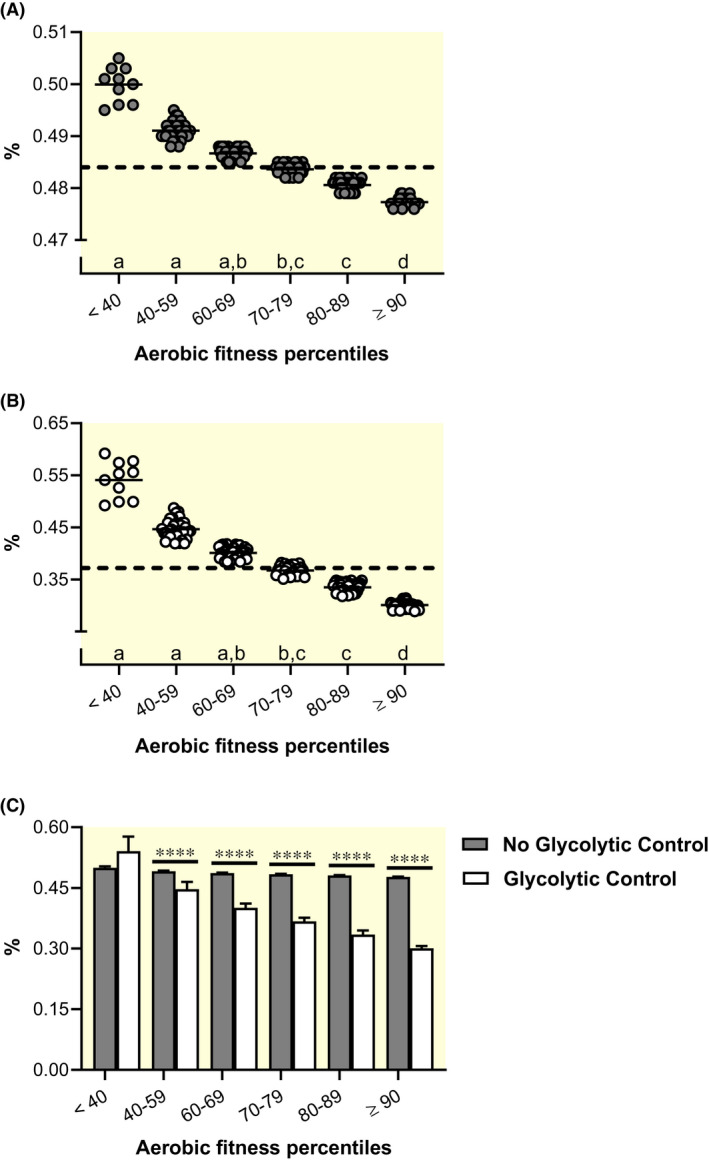
Excess respiratory potential above that determined at maximal whole‐body exercise efforts. Excess respiratory potential was determined as the skeletal muscle respiratory rate at maximal whole‐body cycling efforts relative to temperature‐controlled ex vivo respiratory states representing well‐coupled (P) rates of oxidative phosphorylation (^TEMP^OXPHOS*_p_*) and ^TEMP^OXPHOS*_p_* considering the repressive influence of glycolytic energetics on cellular respiration (^GLYC+TEMP^OXPHOS*_p_*).^76^, ^77^ Individual values and mean (bar) for excess respiratory potential across aerobic fitness percentile subgroups are shown relative to ^TEMP^OXPHOS*_p_* (A) and ^GLYC+TEMP^OXPHOS*_p_* (B) with dashed lines representing respective total group means. Within subgroup comparisons of excess respiratory potential when determined from ^TEMP^OXPHOS*_p_* and ^GLYC+TEMP^OXPHOS*_p_* are presented in C (error bars show SD). Excess respiratory potential and aerobic fitness were analysed using a non‐parametric ANOVA (Kruskal‐Wallis test) with main effects evaluated using Dunn's multiple‐comparison test to control type I error. Different letters represent significant differences across subgroups (*P* < .05) and **** indicates difference *P* < .0001

Aerobic energetic potential of skeletal muscle (^TEMP^OXPHOS*_p_*) above that achieved at CE_MAX_, referred to as excess respiratory potential henceforth, was determined for the collective group (n = 211). There is a main effect for aerobic fitness on excess respiratory potential. Excess potentials specific to ^TEMP^OXPHOS*_p_* (Kruskal‐Wallis statistic = 202.0, *P* < .0001) and ^GLYC+TEMP^OXPHOS*_p_* (Kruskal‐Wallis statistic = 201.6, *P* < .0001) are shown in Figure 5A,B respectively. Accordingly, there is also a main effect of glycolytic repression on excess respiratory potential (Kruskal‐Wallis statistic = 414.4, *P* < .0001) with collective means for excess respiratory capacities of 48.4% (max to min range of 2.9%) for ^TEMP^OXPHOS*_p_* and 36.4% (max to min range of 30.3%) for ^GLYC+TEMP^OXPHOS*_p_*. There is no difference between excess respiratory potential with and without glycolytic control in the least fit group (<40th percentile). However, all other sample groups representing more aerobically fit individuals exhibit statistically significant differences in excess respiratory potential when accounting for glycolytic influence on respiration (Figure 5C).

#### 2.3.1 Temperature‐corrected HRR‐derived excess respiratory potential control

Measures of ^TEMP^OXPHOS*_p_* and ^GLYC+TEMP^OXPHOS*_p_* were adjusted for excess respiratory potential and again compared to complementary in vivo measures. Wilcoxon signed‐rank tests comparing excess respiratory potential controlled (ERP) OCR (117.8 vs 117.8 pmol mg^−1^ s^−1^ and 181.2 vs 181.1 mL kg^−1^ min^−1^), SOR (1.98 vs 1.98 g min^−1^ 7.93 vs 7.91 vs kcal min^−1^) and APR (0.640 vs 0.640 mmol kg^−1^ s^−1^ and 40.3 vs 40.3 mmol/L min^−1^) between ^ERP‐TEMP^OXPHOS*_p_* and ^ERP‐GLYC+TEMP^OXPHOS*_p_* respectively, showed no differences between groups (*P* ≥ .3692, n = 211). Thus, just ^ERP‐TEMP^OXPHOS*_p_* values are analysed and reported.

**FIGURE 6:**
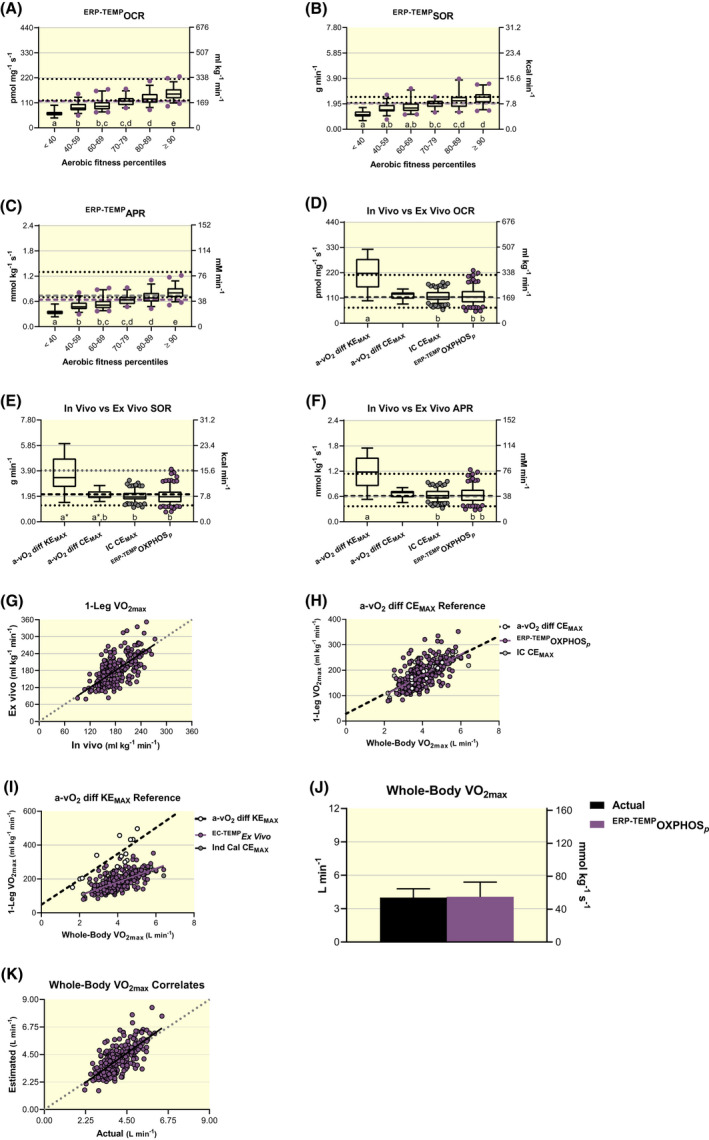
Excess respiratory potential (ERP)‐corrected and temperature‐controlled (TEMP) high‐resolution respirometry (HRR)‐derived rates of oxygen consumption (OCR), substrate oxidation (SOR) and ATP production (APR) with permeabilized human skeletal muscle (ex vivo, n = 211) when compared to indirect calorimetry (IC, n = 211), arteriovenous oxygen difference (a‐vO_2_ diff) during maximal knee extension (KE_MAX_, n = 13) and whole‐body cycling exercise (CE_MAX_, n = 11), and ^31^P magnetic resonance spectroscopy (^31^P MRS, n = 32) methodologies (in vivo). Box and 95% confidence interval‐whisker plots across aerobic fitness percentile subgroups with a purple‐dashed line identifying total group mean, A‐C. Different letters represent significant differences across subgroups (*P* < .05). Excess respiratory‐controlled and temperature‐corrected ex vivo respiratory states represent well‐coupled (P) rates of oxidative phosphorylation (^ERP‐TEMP^OXPHOS*_p_*). Respiratory states and aerobic fitness were analysed using one‐way analysis of variance (ANOVA) assuming Gaussian distribution of residuals. A non‐parametric one‐way ANOVA (Kruskal‐Wallis test) was instead used once this assumption was violated. Significant main effects were evaluated using Bonferroni's or Dunn's multiple‐comparison test respectively, to control type I error. For reference: Average in vivo OCR obtained via a‐vO_2_ diff during CE_MAX_ (184.7 mL kg^−1^ min^−1^; lower dotted line)^36^, ^67^ and a‐vO_2_ diff during KE_MAX_ (328.9 mL kg^−1^ min^−1^; upper dotted line),^28^, ^75^ A; one‐leg estimates of carbohydrate (CHO)‐specific respiration at maximal cycling efforts from moderately active individuals (2.02 g min^−1^; lower dotted line) and professional endurance athletes (2.48 g min^−1^; upper dotted line),^84^ B; and long‐standing estimates of maximal APR derived from CHO‐specific respiration (lower dotted line) and glycolysis (upper dotted line) of 0.70 and 1.3 mmol kg^−1^ s^−1^ respectively.^57^, ^58^ C. The grey‐dashed line in C shows the total group APR mean when adding estimated glycolytic and oxidative energetic contributions at maximal exercise, 0.73 mmol kg^−1^ s^−1^. Box and 95% confidence interval‐whisker plots across methodologies comparing OCR, D; SOR, E; and APR, F, with lower dotted, middle dashed and upper dotted lines representing minimum, mean and maximum ^31^P MRS‐derived values from quadricep muscle during exercise across 32 studies respectively, previously reviewed (*data extracted from figure 9D in reference*).^80^ Different letters represent significant differences across methodologies (*P* < .05) and *indicates 0.1065 ≤ *P* ≤ .1088 across respective methodologies. Representative measures of respiratory control, metabolic flexibility and energetics across methodologies were analysed using a non‐parametric ANOVA (Kruskal‐Wallis test) and main effects evaluated with Dunn's multiple‐comparison test to control type I error. Paired ex vivo to in vivo (IC) estimates of maximal rates of oxygen consumption (VO_2max_) for one leg at CE_MAX_, G; relationships between whole‐body and one‐leg VO_2max_ correlates estimated from HRR‐ and IC‐derived values compared to direct a‐vO_2_ diff assessments during CE_MAX_ and KE_MAX_ in H and I respectively; and paired ex vivo‐derived estimates relative to direct in vivo (IC) assessments of whole‐body VO_2max_, J. Simple linear regression analyses were used to evaluate relationships and comparisons between respective regression lines were evaluated as significant at *P* ≤ .01 to control for type 1 error. Actual IC‐assessed measures of whole‐body VO_2max_ were compared to ^ERP‐TEMP^OXPHOS*_p_*‐derived estimates with repeated measures ANOVA and post hoc pair‐wise evaluations with Bonferroni's multiple‐comparison test to control type I error, K

Descriptive statistics are reported in Table S4 and subgroup data separated by aerobic fitness percentiles are displayed in Figure 6A‐C. There is a main effect of aerobic fitness on ^ERP‐TEMP^OCR (*F* ≥ 39.4, *P* < .0001), ^ERP‐TEMP^SOR (Kruskal‐Wallis statistic ≥78.0, *P* < .0001) and ^ERP‐TEMP^APR (*F* = 39.4, *P* < .0001). Now, ^ERP‐TEMP^SOR (g min^−1^) appear comparable to representative and fitness‐matched rates of CHO oxidation determined with IC (Figure 6B). Specifically, a group of moderately active individuals (n = 20, 40 years and VO_2max_ of 49.6 mL kg^−1^ min^−1^) and professional endurance athletes (n = 22, 26.8 years and VO_2max_ of 74.1 mL kg^−1^ min^−1^) presented with single‐leg average CHO‐specific oxidation rates of 2.02 and 2.48 g min^−1^ respectively,^84^ assuming that working muscle is responsible for ~80% of whole‐body oxidation at CE_MAX_. Those rates compare favourably to respective aerobic fitness‐matched ^ERP‐TEMP^SOR mean ± SD of 2.20 ± 0.61 g min^−1^ (80‐89th percentile) and 2.40 ± 0.49 g min^−1^ (≥90th percentile; Figure 6B). Additionally, estimated rates of ^ERP‐TEMP^APR (0.64 mmol kg^−1^ s^−1^) are closer to traditionally espoused rates of aerobic CHO‐driven ATP synthesis (0.70 mmol kg^−1^ s^−1^)^57^, ^58^ (Figure 6C). It should be noted that the average (n = 211) glycolytic + oxidative APR at CE_MAX_ was calculated as 0.73 mmol kg^−1^ s^−1^ (see grey dashed line in Figure 6C).

There are also main effects of methodology used to calculate OCR (Kruskal‐Wallis statistic ≥24.2, *P* < .0001), SOR (Kruskal‐Wallis statistic = 22.8, *P* < .0001) and APR (Kruskal‐Wallis statistic = 24.3, *P* < .0001) when comparing ex vivo ^ERP‐TEMP^HRR‐derived values to in vivo paired IC and complementary a‐vO_2_ diff‐derived measures (Figure 6D‐F). However, ex vivo determined ^ERP‐TEMP^OCR (*Z* ≤ 0.69, *P* > .9999), ^ERP‐TEMP^SOR (*Z* ≤ 0.91, *P* > .9999) and ^ERP‐TEMP^APR (*Z* ≤ 0.70, *P* > .9999) are equivalent to paired and corresponding in vivo IC and a‐vO_2_ diff at CE_MAX_ respectively. For example, single‐leg OCR for a‐vO_2_ diff at CE_MAX_, IC at CE_MAX_ and ^ERP‐TEMP^OXPHOS*_p_* are 184.7, 181.1 and 181.2 mL kg^−1^ mL^−1^ respectively (Figure 6D). Importantly, ex vivo ^ERP‐TEMP^HRR‐derived values also resemble complementary measures obtained with ^31^P MRS. For example, average APR determined by a‐vO_2_ diff at CE_MAX_, IC at CE_MAX_, ^ERP‐TEMP^OXPHOS*_p_* and average ^31^P MRS estimates from quadricep muscle during exercise^80^ are 41.0, 40.3, 40.3 and ~38.4 mmol/L min^−1^ respectively (Figure 6F). Alternatively, measures of OCR (*Z* ≥ 2.93, *P* ≤.034), SOR (*Z* ≥ 4.35, *P* ≤.0001) and APR (*Z* ≥ 2.94, *P* ≤.0333) determined from a‐vO_2_ diff at KE_MAX_ are higher than all other methodologies (Figure 6D‐F) apart from SOR comparisons between a‐vO_2_ diff at CE_MAX_ and KE_MAX_ (*Z* = 2.55, *P* ≤ 1.088).

Paired ex vivo ^ERP‐TEMP^OXPHOS*_p_* and in vivo IC‐derived estimates of maximal 1‐leg OCR statistically resemble a perfect linear relationship (slope: F < 0.01, *P* = .9907; and y‐intercept: *F* < 0.01, *P* = .9960; Figure 6G). Regression lines for maximal 1‐leg OCR (mL kg^−1^ min^−1^) relative to whole‐body VO_2max_ (L min^−1^) also do not differ (slope: *F* = 0.01, *P* = .9042; and y‐intercept: *F* = 0.04, *P* = .8509) when comparing ^ERP‐TEMP^OXPHOS*_p_* to a‐vO_2_ diff during CE_MAX_ (Figure 6H). Only slopes comparing ^ERP‐TEMP^OXPHOS*_p_* to a‐vO_2_ diff values collected during KE_MAX_ are different (*F* = 9.42, *P* = .0024; Figure 6I).

Wilcoxon signed‐rank tests identify no effect of methodology on absolute (*P* = .5150; Figure 6J) or relative (*P* = .4823; data not shown) VO_2max_ determination when comparing actual IC‐derived measures to ^ERP‐TEMP^OXPHOS*_p_* extrapolations. Paired ^ERP‐TEMP^HRR‐ and IC‐derived VO_2max_ correlates do not statistically differ from a perfect relationship (slope: 0.75, *P* = .3884; and y‐intercept: *F* = 1.77, *P* = .1842; Figure 6K).

Collectively, correcting temperature and controlling for excess respiratory potential transforms standard HRR‐derived measures of OCR, SOR and APR from permeabilized human skeletal muscle samples to resemble complementary in vivo measures obtained during CE_MAX_.

### 2.4 Flux control ratios

There are main effects of aerobic fitness on the flux control ratios comparing FAO*_p_* to OXPHOS*_p_* (FAO*_p_* OXPHOS*_p_* ^−1^: *F* = 16.4, *P* < .0001; and ^TEMP^FAO*_p_* ^TEMP^OXPHOS*_p_* ^−1^: *F* = 11.1, *P* < .0001) with those individuals classified in the ≤90th aerobic fitness percentile as higher than all other subgroups (*t* ≥ 4.7, *P* < .0001 and *t* ≥ 3.8, *P* ≤ .0034 respectively; Figure S4,A,B). Alternatively, there is no main effect of aerobic fitness on the flux control ratio comparing OXPHOS*_p_* to ETS (Kruskal‐Wallis statistic = 10.3, *P* = .0676) regardless of temperature control (OXPHOS*_p_* ETS^−1^ = ^TEMP^OXPHOS*_p_* ^TEMP^ETS^−1^; Figure S4C).

## 3 DISCUSSION

This study sought to: (i) Provide a statistically robust reference for measures of mitochondrial function in relation to oxygen consumption rates (OCR), substrate oxidation rates (SOR) and ATP production rates (APR) obtained using standardized HRR methodologies (ie physiological temperatures of 37°C and high respiratory chamber oxygen concentrations of ~250 to 500 μmol/L) with permeabilized human skeletal muscle samples; (ii) Compare these ex vivo reference values to analogous measures collected with alternative in vivo methodologies (ie IC, a‐vO_2_ diff and/or ^31^P MRS) and (iii) Attempt to resolve differences across complementary ex vivo and in vivo methodologies as necessary.

As per the first study aim, reference values of OCR, SOR and APR for HRR‐derived measures of FAO*_p_* (n = 189), OXPHOS*_p_* (n = 211) and ETS (n = 187) collected under standard conditions are reported in Tables 2, 3, 4 respectively. These values serve as an accessible reference for HRR‐derived indices of mitochondrial function from permeabilized human skeletal muscle under standardized conditions relative to a population (28.1 ± 6.1 years; 53.7 ± 11.3 VO_2max_ mL kg^−1^ min^−1^) free of heart and/or metabolic disease (Table 1).

As per the second study aim, these HRR reference values, obtained under standardized conditions across various laboratories, research groups and technicians, are lower en masse than corresponding values collected with in vivo methodologies, including IC, a‐vO_2_ diff and ^31^P MRS (Figures 1 and 2).

As per the third study aim, correcting respiratory measures to reflect approximate mitochondrial temperatures 10°C above skeletal muscle temperature at maximal exercise efforts, ~50°C,^82^ transforms standardized HRR‐derived values to those that closely resemble certain corresponding in vivo measures. Temperature‐corrected FAO*_p_* (^TEMP^FAO*_p_*) SOR compare favourably to fitness‐matched maximal rates fat oxidation (MFO; g min^−1^), as assessed with IC methodologies^53^, ^54^, ^55^, ^56^ (Figure 3B) and the collective group APR mean (Table S1) corresponds to traditionally reported rates of fat‐specific ATP production (Figure 3C).^57^, ^58^ Temperature‐corrected OXPHOS*_p_* (^TEMP^OXPHOS*_p_*) values are statistically comparable to fitness‐matched measures determined from a‐vO_2_ diff during maximal efforts of normoxic one‐legged knee extension exercise (KE_MAX_; Figures 3D and 4A‐C,F, & Table S2).^28^, ^75^ Alternatively, ^TEMP^OXPHOS*_p_*‐derived OCR, SOR and APR are higher than complementary in vivo (IC and a‐vO_2_ diff) measures obtained during maximal efforts of normoxic two‐legged cycling exercise (CE_MAX_; Figures 3D and 4A‐C,E, & Table S2).^36^, ^67^ Thus, the idea of a skeletal muscle respiratory potential in excess of that achieved during maximal whole‐body exercise efforts (eg CE_MAX_) is supported with and without considering the repressive influence of glycolytic ATP production on cellular respiration^76^, ^77^ (Figure 5A,B). Controlling for this ostensible excess respiratory potential (ERP) above that achieved during whole‐body maximal exercise efforts transforms temperature‐corrected HRR‐derived respiratory values (^ERP‐TEMP^OXPHOS*_p_*) to resemble analogous fitness‐matched in vivo measures collected with IC and a‐vO_2_ diff at CE_MAX_ and ^31^P MRS obtained from quadricep muscle during exercise^80^ (Figure 6A,B,D‐H,J,K).

### 3.1 Aim 1: HRR reference values with healthy permeabilized human skeletal muscle

Establishing biologically relevant references for ‘healthy’ human respiratory control, metabolism and bioenergetics across the lifespan is necessary to discern, interpret and combat the ostensible dysfunction commonly referenced as somewhat responsible for a myriad of human diseases and disorders. The predominance of mitochondrial dysfunction in the aetiology of most prevalent non‐communicable diseases is generally accepted and empirically supported as recently reviewed by Diaz‐Vegas et al.^86^ For better or worse (beneficial or detrimental), skeletal muscle and mitochondrial function also appear to ferry a considerable degree of biological function into senescence.^87^ Yet, we are largely unable to discern healthy from unhealthy tissue‐specific values of respiratory control, metabolic flexibility and bioenergetic potential. More worrying, we struggle in our collective ability to differentiate legitimate biological values from those that may be heavily influenced from the many pitfalls of unintended methodological oversight. The work presented here was completed with the general goal of advancing our collective knowledge regarding *healthy* indices of *mitochondrial function* in human skeletal muscle and improving our ability to scrutinize HRR‐derived data collected from permeabilized human skeletal muscle samples alongside complementary research utilizing alternative in vivo techniques. To achieve this goal, a ‘healthy’ population had to be identified for reference.

The collective sample population examined in this study had an average VO_2max_ of 53.7 mL kg^−1^ min^−1^ or 4.0 L min^−1^, which averages as slightly above the ~70th aerobic fitness percentile when controlling for age (28.1 years) and sex (13.7% to 86.3% female‐to‐male data representation respectively) as per ACSM guidelines^52^ (Table 1). This verifies our intended design. Our collective group OXPHOS*_p_* mean and SD is 94.9 ± 24.7 pmol mg^−1^ s^−1^ (n = 211; CV = 26.0%; Table 3). Interindividual CV increased by an average of 0.7 and 2.8% when temperature correcting FAO*_p_* (36.9%, Table 2, to 37.6%, Table S1) and OXPHOS*_p_* (26.0%, Table 3, to 28.8%, Table S2) values respectively. Interindividual variance across participants with standardized HRR assessments on permeabilized human skeletal muscle samples appears equivalent to other skeletal muscle characteristics often used to ascribe skeletal muscle ‘health’ such as muscle fibre cross‐sectional area, fibre‐type distribution percentages, enzyme activities (mean CV for 6 fibre‐type and 6 enzymatic measures across female, n = 203, and male, n = 215, participants of 33.7% with min‐max of 21%‐72%)^88^ as well as ^31^P MRS during exercise (mean CV ~ 31% for data presented in figure 9D specific to quadriceps analyses).^80^ Interindividual variance with IC and a‐vO_2_ diff methodologies resembles the variance of aerobic fitness across respective experimental groups (CVs for absolute VO_2max_ and average indices of mitochondrial function for IC at CE_max_, a‐vO_2_ diff at CE_max_ and a‐vO_2_ diff at KE_max_ are 19.7 and 19.3%, 15.1 and 17% and 31 and 34% respectively). We also assessed within‐participant or intra‐individual variability using our largest data set collected by the same HRR technician. The intra‐individual CV (duplicate or more measures obtained from the same skeletal muscle biopsy) for OXPHOS*_p_* is 14.8% (n = 89). This within‐participant variation agrees with previous reports of 15.2% (n = 25)^89^ and 15.3% (n = 68).^90^

The origination of data used for analysis and presentation in this study consists of ~75% (n ≤ 159) that were amassed from individuals participating in our own research and ~25% (n ≤ 52) from respective group means published across the literature,^29^, ^30^, ^31^, ^32^, ^33^, ^34^, ^35^, ^36^, ^37^, ^38^, ^39^, ^40^, ^41^, ^42^, ^43^, ^44^, ^45^, ^46^, ^47^, ^48^, ^49^, ^50^, ^51^ as described in our methods. Collectively, these data are derived from 831 study participants across research published throughout the past decade (March 2009 to November 2019). Respiratory measures assessed in this study were obtained from several different research laboratories consisting of various researchers and/or technicians completing all relating HRR methodology, ie skeletal muscle biopsy collection, preparation and storage of necessary chemicals and media, skeletal muscle permeabilization, respirometric analyses and statistical evaluations. This is all important to consider when interpreting these results to other published findings, as the current study has compiled the largest collective human sample size across the most diverse research settings to date.

The importance in establishing some biologically relevant standard agreement across the field for HRR‐derived respiratory measures when using permeabilized skeletal muscle samples can be appreciated when comparing two different studies examining aspects of variability with standardized HRR on permeabilized human skeletal muscle.^89^, ^90^ Cardinale et al (2018) reports a mean ± SD OXPHOS*_p_* of 69.2 ± 17.0 pmol mg^−1^ s^−1^ from a group (n = 25) of “well‐trained” (no apparent report of VO_2max_ to our best discernment) young (24.7 ± 4.5 years) men,^89^ whereas Jacques et al (2020) reports a mean ± SD OXPHOS*_p_* of 123.1 ± 37.5 pmol mg^−1^ s^−1^ from a group (n = 68) of “moderately‐trained” healthy participants (VO_2max_ = ~3.9 L min^−1^ ^91^; age = 31.4 ± 8.2 years).^90^ The discrepancy between these appropriately powered human studies examining standardized HRR‐derived rates of skeletal muscle respiration across comparable sample groups (sex, age and fitness) is concerning. The current study failed to identify as sizable a discrepancy for similarly powered comparisons between subgroups representing individuals that are categorized in 40‐59th (n = 34; 81.1 ± 17.9 pmol mg^−1^ s^−1^) and ≥90th (n = 56; 115.5 ± 21.4 pmol mg^−1^ s^−1^) aerobic fitness percentiles despite significant differences in whole‐body VO_2max_ (43.1 vs 68.8 mL kg^−1^ min^−1^ respectively). Empirically supported assumptions would rightly anticipate that mitochondrial characteristics are higher in those that are comparatively more exercise trained and/or aerobically fit while similar across groups that resemble one another.^92^, ^93^, ^94^, ^95^ Standardized HRR‐derived respiratory rates do not currently benefit from any semblance of a validated source reference of which could aid researchers and clinicians in scrutinizing the appropriate biological context of their measures. In addition to the reference values identified in this study (Tables 2, 3, 4), we also describe and validate a method to estimate single‐leg rates of oxygen consumptions at maximal cycling efforts using common indirect calorimetry methodology. This provides an internal cross‐methodological control for future studies utilizing HRR on human skeletal muscle assessing the upper limits of mitochondrial respiratory control.

It is imperative that continued research involving skeletal muscle mitochondrial assessments stand somewhat responsible for discerning their own results as contextually relevant to minimize the influence of unintended methodological oversight on our collective progression in all related fields of study. Here, we describe an approach for future research to consider. Controlling for the potentially confounding methodological effect of chamber temperature (typically assessed 37°C) and acknowledging the influence of parallel non‐aerobic metabolism on cellular energetics appear to improve upon the biological relevance of HRR.

### 3.2 Aim 2: comparing HRR (ex vivo) to analogous in vivo methodologies

The collective 211 HRR‐derived values included in the current study, amassed across 831 study participants from standardized HRR protocols, show that OXPHOS*_p_*‐specific OCR, SOR and APR are lower than analogous measures collected from paired IC‐derived estimates as well as comparative a‐vO_2_ diff and/or ^31^P MRS methodologies (Figures 1 and 2). Importantly, and as stated in the previous section, IC‐derived estimates of maximal leg OCR, SOR and APR from whole‐body VO_2max_ (L min^−1^) compare favourably with like measures from a‐vO_2_ diff and ^31^P MRS methodologies. While this is the first study to compare equivalent indices of skeletal muscle mitochondrial function across standardized HRR protocols as well as IC, a‐vO_2_ diff and ^31^P MRS methodologies, our collective understanding that HRR results in lower values than complimentary in vivo methodologies have been acknowledged for at least two decades.

In 2001, Rasmussen et al reported that maximal state 3 respiration derived from isolated human skeletal muscle mitochondria, temperature corrected to 38°C and extrapolated to whole‐muscle estimates of quadricep VO_2max_, were lower than direct a‐vO_2_ diff measures during KE_max_ (see figure 1F in reference).^79^ In a 2009 review, Dr Erich Gnaiger (Oroboros Instruments CEO) affirmed that HRR with well‐coupled mitochondrial preparations fell short of a‐vO_2_ diff assessments during KE_max_ even when temperature correcting HRR assessments from 37 to 38°C; “*Respiratory capacities measured in well coupled mitochondrial preparations, therefore, fall short of explaining the high respiratory capacity of human skeletal muscle in vivo, even when taking into account the temperature increase from 37 to 38°C and corresponding stimulation of respiration by approximately 7%*.”.^26^ Boushel *et al* (2011) temperature‐corrected HRR values from permeabilized skeletal muscle samples to femoral venous temperatures at maximal cycling efforts ranging from 39 to 39.7°C, resulting in a mean OXPHOS*_p_* of ~115 pmol mg^−1^ s^−1^ across a group (age 33 years) of men (n = 5) and women (n = 4) with a mean VO_2max_ of 3.46 L min^−1^ (~45.5 mL kg^−1^ min^−1^).^65^ This respiratory value is ~40% higher than the standardized and ~40% lower than the temperature‐corrected rates determined in the present study when controlling for aerobic fitness and sex (60‐69th aerobic fitness percentile; 84.1 ± 21.7 pmol mg^−1^ s^−1^, Figure 1D; and 196.9 ± 52.8 pmol mg^−1^ s^−1^, Figure 3D respectively). Gifford *et al* (2016)‐derived OXPHOS*_p_* at 37°C with permeabilized human skeletal muscle “*and then mathematically adjusted, based on a Q_10_ of 2 (multiplication factor for O_2_ consumption at a 10°C difference), to yield predicted values at 38°C*” to obtain reported group means of approximately 238.1 pmol mg^−1^ s^−1^ from 10 untrained male participants (age 25 year; 2.9 L min^−1^, 38 mL kg^−1^ min^−1^) and 486.6 pmol mg^−1^ s^−1^ from 10 trained male participants (age 24 year; 4.1 L min^−1^, 59 mL kg^−1^ min^−1^).^28^ Thus, it is clear that standardized HRR‐derived measures with human skeletal muscle samples at 37°C are lower than analogous in vivo assessments during exercise and some degree of temperature correction is necessary to improve upon the biological relevance of HRR‐derived OCR, SOR and APR.

### 3.3 Aim 3a: temperature correcting standardized HRR measures with permeabilized human skeletal muscle

Consideration of temperature control over human metabolism and bioenergetics is critical. For example, exercise training improvements in HRR‐derived skeletal muscle respiration and efficiency are apparent at exercising (40°C) but not resting (35°C) skeletal muscle temperatures.^48^ These findings provide basic context to the concept of compromised biological nuance with unintentional yet ubiquitous methodological oversight. Therefore, tissue‐specific, and possibly mitochondrial‐specific, temperatures should be considered to improve upon the biological relevance of HRR assessments. However, a divergence of empirical and theoretical findings over the heterogeneous nature of cellular thermodynamics and subsequent cellular temperature gradient(s) has resulted in a contentious debate that currently obscures our understanding of relevant cellular and/or mitochondrial temperature spectrums for indisputable consideration.

A recent review summarizing empirical and theoretical findings surrounding the debate of accurate in vivo mitochondrial temperatures identifies 10 studies that report an increase in temperature with mitochondrial respiratory uncoupling, and 5 of those studies are reported to identify temperature heterogeneity in the organelle when using fluorescent thermosensors to study mitochondrial heating and temperature (*see Table 1 in reference*).^96^ Of these studies, Chretien *et al* (2018) notably provided seminal evidence to suggest that several components of the electron transport system function optimally at temperatures reaching over 50°C, or ~10°C higher than the encompassing cell when studying HEK 293 cells and primary skin fibroblasts.^82^ These findings have since been verified in HeLa cell lines.^83^ Several issues have been raised in opposition of these findings, such as: (i) methodological concerns specific to research utilizing fluorescent probes for determination of cellular temperatures that includes possibly confounding influence(s) of the surrounding environment (ie pH, reactive oxygen species, membrane potential, viscosity and ionic strength); (ii) thermodynamic modelling of the cell describing the so‐called “10^−5^ gap” theory that renders intracellular temperature gradients as all but impossible and (iii) biological improbabilities of such high in vivo temperatures that would challenge human biological function as we understand it.^96^, ^97^, ^98^ Thus, considerations for appropriate temperature corrections range from a minimum that reflects the cellular temperature specific to the tissue being analysed when taking into account the metabolic state also being measures (ie basal vs maximal metabolic states) to a maximum of 10°C above that minimum value. As noted previously, temperature corrections used to reflect the temperature of the exercising muscle (eg 38°C) are still lower than in vivo methodologies identify.^26^, ^79^ Slightly higher‐temperature corrections to reflect skeletal muscle temperatures during maximal exercise efforts ~39 to 40°C have also been used for previous mitochondrial research.^48^, ^65^ We find that this accompanying increase in OXPHOS*_p_* of ~19% statistically resembles IC estimates and direct a‐vO_2_ diff measures at CE_max_ (175.2 vs 181.1 and 184.7 mL kg^−1^ min^−1^ respectively; Kruskal‐Wallis statistic ≤1.9, *P* ≥ .3334), unlike the excess respiratory potential that has previously been reported with respirometric correction to 39‐39.7°C.^65^ Moreover, temperature correcting respiratory values to ~39 to 40°C still results in OCR lower than observed with a‐vO_2_ diff at KE_max_ (175.2 vs 328.9 mL kg^−1^ min^−1^ respectively; Kruskal‐Wallis statistic = 5.2, *P* < .0001). Given that blood flow and rates of skeletal muscle oxygen consumption have repeatedly been shown as higher during maximal isolated vs whole‐body exercise efforts (eg KE_max_ vs CE_max_) and assuming that respiration during maximal isolated exercise efforts remains well‐coupled to ATP production, temperature correcting standardized HRR values obtained from permeabilized human skeletal muscle appears to require corrections above skeletal muscle temperatures at maximal exercise efforts. Thus, the current study examined how temperature correcting standardized HRR values to 10°C above respective cellular temperatures influences measures reflecting respiratory control (OCR), metabolic flexibility (SOR) and bioenergetics (APR), which is the maximal temperature correction that currently entertains empirical support,^82^, ^83^ albeit contested.

Correcting respiratory measures to reflect temperatures 10°C higher than skeletal muscle during maximal exercise efforts transformed OXPHOS*_p_* values to statistically resemble complimentary measures obtained from a‐vO_2_ diff during KE_MAX_ ^28^, ^75^ but are higher than those acquired from a‐vO_2_ diff during CE_MAX_ ^36^, ^67^ (Figures 3 and 4). These findings confirm previous claims that temperature‐corrected HRR OXPHOS*_p_* values demonstrate an excess respiratory potential above that required during CE_MAX_.^65^ Taking the difference of temperature corrections into account, excess respiratory potential respective to maximal whole‐body aerobic power is more likely ~48% to 49% (Figure 5A) opposed to the ~38% previously identified^65^ without considering respiratory attenuation by glycolytically derived ATP. However, these findings do not support the claim of excess respiratory potential respective to OCR determined during KE_MAX_ (Figure 4A). Reported HRR‐derived OXPHOS*_p_* OCRs of ~364 mL kg^−1^ min^−1^ and ~744 mL kg^−1^ min^−1^ from sample populations equivalent to <40th and 80‐89th aerobic fitness percentile subgroups most likely represent some error in respiratory temperature correction, as these values are ~60% and 100% higher than fitness matched ^TEMP^OXPHOS*_p_* values respectively (286.4 and 369.1 mL kg^−1^ min^−1^; Figure 3D). Temperature correction is not the only factor that should be considered when interpreting HRR‐derived measures of respiratory control, metabolic flexibility or bioenergetics into an appropriate biological context. Accurate quantification of functional mitochondrial characteristics should also account for the repressive influence of glycolytic substrate‐level phosphorylation on oxidative phosphorylation for a given metabolic state.

### 3.4 Aim 3b: glycolytic considerations influence interpretation of standardized HRR measures with permeabilized human skeletal muscle

Glycolytically derived ATP that alters the cellular adenylate equilibrium by increasing the ratio of ATP to ADP + P_i_ and subsequent ΔG_ATP_ creates more back pressure on ATP synthase and reduces the rate of ATP production,^77^ which has been demonstrated.^76^ We estimated glycolytic contributions to maximal rates of ATP production during whole‐body CE_MAX_ to determined excess respiratory capacities attenuated by glycolytic restraint and compare against raw excess respiratory potential with no glycolytic influence (Figure 5). Our calculation of glycolytic contribution compared favourably albeit ~1% higher to a previously published method estimating glycolytic contributions during maximal incremental cycling efforts^99^ (Figure S6B). IC‐derived estimates of aerobic APR, which are similar to a‐vO_2_ diff‐^36^, ^67^ and ^31^P MRS‐derived^80^ estimates (Figures 2A‐C, 4A‐C and 6D‐F), are lower than traditional claims of CHO‐driven respiratory APR (0.64 vs 0.70 mmol kg^−1^ s^−1^).^57^, ^58^ Yet, adding glycolytic‐estimated rates of APR to those oxidative estimates, regardless of glycolytic derivation method, combine to resemble previous claims of mitochondrial‐specific ATP production averages (0.73 mmol kg^−1^ s^−1^; Figure 6C). Energetic homeostasis is dependent on complementary aerobic and non‐aerobic means of energy transfer in effort to maintain intracellular ATP concentrations. Complete interpretations of human metabolic flexibility require that integrative efforts of respective cellular energy systems be accounted. The collective results in this study demonstrate the importance of considering corresponding glycolytic and respiratory rates. There is no observable difference between excess respiratory potential with and without glycolytic control in the least fit subgroup (<40th percentile), whereas all other subgroups representing more aerobically fit individuals (>40th percentile) exhibit statistically significant differences in excess potential when accounting for glycolytic respiratory attenuation (Figure 5C).

Hyperoxia has been shown to improve maximal work rates^60^, ^61^, ^100^ and PCr recovery kinetics^101^ in trained and relatively fit individuals, whereas maximal work rates^102^ and PCr recovery kinetics^103^ are not improved by hyperoxia in less fit sedentary individuals. This has been interpreted as an ostensible excess respiratory potential in fit individuals that is not apparent in unfit counterparts. Alternatively, the findings presented in this study suggest that excess respiratory potential is relatively higher (% of maximal respiratory potential) in unfit individuals and progressively declines with improving fitness (Figure 5). Considering these observations, we postulate that differential effects of hyperoxia on skeletal muscle bioenergetics between trained and sedentary counterparts is more likely attributable to hyperoxic influences on the ratio of glycolytic substrate‐level phosphorylation rates to oxidative phosphorylation rates and the resulting myocellular adenylate equilibrium. These findings (Figure 5C) suggest that hyperoxia would not suppress glycolytic rates in untrained sedentary individuals and thus would not delay glycolytic contributions to fatigue‐inducing metabolic by‐product accumulation (eg P_i_ and H^+^)^58^, ^104^, ^105^ or alter the adenylate equilibrium as would occur in somewhat to highly trained individuals. This concept parallels the occurrence of exercise‐induced arterial hypoxemia (EIAH) that is more prevalent in fit individuals^106^ and introduces the idea that one's EIAH may direct skeletal muscle metabolic phenotype and bioenergetic function. It is unlikely that EIAH per se describes divergent influences of hyperoxia on 5‐min steady‐state submaximal plantar flexion exercise between exercise‐trained^101^ and sedentary individuals.^103^ Alternatively, the ratio of glycolytic relative to oxidative skeletal muscle energetic potential adapted to complement EIAH experienced at high‐to‐maximal efforts does theoretically support the differing influences of hyperoxia between those that are fit and unfit even during submaximal activity in which oxygen availability is not limited and hypoxemia is not achieved.

### 3.5 Study limitations, additional methodological considerations with HRR and future directions

As with all research, the findings presented in this study should be interpreted and applied with contestable assumptions inherent to data collection and analysis acknowledged. This research assumes that: mitochondrial temperatures reach 10°C higher than the encompassing cell, 4‐10 mg of permeabilized human skeletal muscle for duplicate measures (typically obtained from the vastus lateralis) is representative of all active skeletal muscle during maximal cycling and knee extension exercise efforts; P‐state respiration with maximal convergent flow of electrons into the Q‐cycle from NADH dehydrogenase and succinate dehydrogenase appropriately simulates maximal in vivo rates of mitochondrial oxidative phosphorylation; the standardized experimental milieu, in general, allows for appropriate determination of maximal respiratory rates; maximal rates of myocellular respiration in vivo are well‐coupled allowing for the use of static P:O ratios in metabolic and bioenergetic calculations; mitochondrial NADH can functionally persist at temperatures ~50°C or that the standard protocol used to measure OXPHOS*_p_* appropriately captures respiratory rates that may be maintained by alternative routes of electron input not included in HRR analyses such as the glycerol phosphate shuttle^107^; the estimations used for oxygen consumption and substrate partitioning throughout the body during maximal exercise efforts are generally accurate for a large sample population in which VO_2max_ varies from 25.6 to 83.5 mL kg^−1^ min^−1^ representing maximal estimated aerobic powers of 228 to 398 W respectively; fat oxidation in active skeletal muscle is negligible during maximal efforts of whole‐body exercise; ΔG_ATP_ = −11.5 kcal mol^−1^; methods for estimating leg muscle mass are accurate; etc. Additionally, this study is only referencing experimental protocols that utilize standardized HRR protocols with permeabilized human skeletal muscle samples. Similar research should be conducted on the relevance of protocols utilizing isolated mitochondrial preparations, HRR protocols that use relatively lower chamber oxygen concentrations with permeabilized fibre samples, data reported relative to dry vs wet weight and HRR protocols that publish respiratory rates with electron input isolated to just one pathway through the electron transport system. All future research bears the burden to continually improve upon our collective ability to interpret the biological relevance of mitochondrial assessments with HRR. These continued efforts are important as no other methodology allows for the analysis or respiratory control, metabolic flexibility and bioenergetics with one small tissue sample. While future efforts will undoubtedly improve upon our methodology, the results presented in this study, especially as they relate to multiple fields of complementary research, should be scrutinized as more than simple coincidence even when accounting for study limitations.

#### 3.5.1 Conclusion

Standardized HRR with permeabilized human skeletal muscle results in measures that are lower than corresponding values collected with in vivo methodologies, including IC, a‐vO_2_ diff and ^31^P MRS. Correcting respiratory measures to reflect approximate mitochondrial temperatures 10°C above skeletal muscle at maximal exercise efforts, ~50°C,^82^ transforms standardized HRR‐derived values to resemble certain corresponding in vivo measures (eg MFO and a‐vO_2_ diff during KE_MAX_) but are higher than other complementary in vivo measures (IC and a‐vO_2_ diff at CE_MAX_). This disparity supports the idea of a skeletal muscle respiratory potential that exceeds what is achieved during maximal whole‐body exercise efforts (eg CE_MAX_). However, consideration of parallel glycolytic energetics is also necessary to fully interpret the biological significance ex vivo‐derived respiratory rates in reference to human metabolic health.

## 4 MATERIALS AND METHODS

### 4.1 Respiratory states

A priori regression analyses using a subset of all data in which complete respiratory analyses were immediately available (n = 89) revealed three specific respiratory states derived from HRR on permeabilized skeletal muscle samples that statistically stand out as more divergent when compared, and thus more related, to relative measures of whole‐body aerobic capacities ranging from 31.7 to 81.9 mL kg^−1^ min^−1^ (Figure S5). Those respiratory states include: (i) maximal state 3 rates of well‐coupled respiration (P) with lipid substrates (octanoyl‐ or palmitoyl‐carnitine) supplying maximal electron input to the Q‐cycle from the electron‐transferring flavoprotein complex with some simultaneous malate‐driven electron input via NADH dehydrogenase, experimentally administered to represent maximal rates of mitochondrial fatty acid oxidation (FAO*_p_*) in skeletal muscle; (ii) P‐state respiration with maximal convergent flow of electrons into the Q‐cycle from NADH dehydrogenase via malate, pyruvate and/or glutamate as well as succinate dehydrogenase via succinate, experimentally administered to represent maximal rates of mitochondrial oxidative phosphorylation (OXPHOS*_p_*) in skeletal muscle and (iii) maximal rates of non‐coupled respiration (E) with analogous electron flow into the Q‐cycle as OXPHOS*_p_*, commonly referred to as the electron transfer state (ETS) and discussed as the respiratory state that is uninhibited by phosphorylative restraint. Also, FAO*_p_* and OXPHOS*_p_* provide the only relatable HRR references for complimentary in vivo measures collected with other methodologies. Accordingly, subsequent analyses conducted focused on these three respiratory states.

### 4.2 Internal data inclusion

Standardized mitochondrial evaluations derived from HRR with permeabilized human skeletal muscle tissue from our research dating back to 2010 were compiled. Datum was identified for analysis if participant age was <50 years, BMI < 35 kg/m^2^, they reported no use of medication(s) that were known or likely to influence human metabolic regulation and they did not present with signs or a medical diagnosis of heart or metabolic disease. Pre‐ and post‐exercise training values were included, whereas only baseline values were included from participants volunteering in studies if experimental treatment(s) altered measures of respiratory control (eg hypoxia^108^).

### 4.3 External data inclusion

A systematic search of the literature was conducted in PubMed, including relevant studies up until July 2020. Studies were included if they: (i) reported values derived from standardized HRR techniques with permeabilized human skeletal muscle samples and respiratory rates were presented in pmol O_2_ per mg wet weight of the sample per second (studies using isolated mitochondria or reporting mass‐specific respiratory measures per dry weight were not considered); (ii) reported data reflecting the OXPHOS*_p_* respiratory state; (iii) reported necessary study participant characteristics, which included age, body mass and maximal rates of whole‐body oxygen consumption (VO_2max_) and (iv) matched inclusion/exclusion criteria for study participants as detailed for internal data inclusion.

### 4.4 External data extraction

Requisite data from each study identified for external data inclusion were gathered, which included OXPHOS*_p_*, age, body mass and VO_2max_. Additional data, including height, BMI, FAO*_p_* and ETS, were included when available. Externally sourced data were extracted using WebPlotDigitizer^109^ (Web Plot Digitizer, v.4.2, 2019, Ankit Rohatgi, https://automeris.io/WebPlotDigitizer, Pacifica, California, USA)^110^, ^111^, ^112^, ^113^ if not presented in table or text. Verification of data extraction accuracy was substantiated using a subset of our own publications^94^, ^108^, ^114^, ^115^ to compare extracted values to the actual measured values (n = 80). Digitizing drift (|digitized data − actual data|/actual data) was <1% (0.85%) and data matching (actual vs digitized correlates) was identified as excellent (*F* = 426 958; *R* ^2^ = 0.9998, 95% CI slope = 0.9926‐0.9987).

### 4.5 Standardized HRR experimental conditions

All respiratory rates included for analysis were derived under standard conditions, ie physiological temperatures of 37°C and high respiratory chamber oxygen concentrations of ~250 to 500 μmol/L to minimize artificial limitations of oxygen supply.^25^, ^26^, ^81^ Oxygen's electronegativity, second to fluorine, establishes the redox gradient governing oxidative phosphorylation^77^ and thus limitations in oxygen availability while conducting respiratory assessments result in artificially diminished rates of respiration.^116^ Published respiratory rates included that were collected at temperatures lower than 37°C^35^, ^36^, ^38^ had been adjusted to 37°C assuming a 10‐degree temperature coefficient (Q_10_) of 2, as later described in 4.9 Temperature Correcting Respiratory Values, or averaged across complementary temperatures at a given time point (35°C and 40°C).^48^

### 4.6 Conversions

#### 4.6.1 Oxygen consumption rates (OCR): pmol mg^−1^ s^−1^ to mL kg^−1^ min^−1^

Conversion from pmol mg^−1^ s^−1^ to mL kg^−1^ min^−1^ adhered to Charles's Law or the Law of Volumes, which states that for a given mass of an ideal gas at constant pressure, the volume is directly proportional to its absolute temperature, assuming a closed system. Thus,

(1) $$V_{1}/T_{1}=V_{2}/T_{2}$$

where *V*_1_ is the molar equivalent of oxygen, 22.4 L per mol, at a standard temperature, *T*_1_ (273 K or 0°C) and *T*_2_ is femoral venous temperature at maximal exercise intensity. Femoral venous temperature was determined by the change in oxygen consumption (L min^−1^) from rest to maximal exercise^66^:

(2) $$T_{2}=0.1065\times \Delta {\text{VO}}_{2}^{2}‐0.0214\times \Delta {\text{VO}}_{2}+37.361$$

Resting oxygen consumption was estimated as described by Dehmer et al^117^:

(3) $${\text{VO}}_{2\text{rest}}\left(\text{mL}/\text{min}\right)=125\left(\text{mL}\times {\left(\text{min}\times m^{2}\right)}^{‐1}\right)\times \text{body surface area}\left(\text{BSA},\;m^{2}\right)$$

BSA calculated according to the formula of Dubois & Dubois^118^:

(4) $$\text{BSA}\left(m^{2}\right)=0.007184\times \text{weight}{\left(\text{kg}\right)}^{0.425}\times \text{height}{\left(\text{cm}\right)}^{0.725}$$

Mean femoral venous temperature for the collective sample group analysed (n = 211) was 39.5°C with a range from 38.3 to 42.1°C. Therefore, *V*_2_ ranged from 25.5 to 25.9 L with a mean of 25.6 L. This compares to the more commonly referenced range from 25.4 to 25.5 L assuming average skeletal muscle temperatures of 37‐38°C during KE_MAX_ ^119^ or 25.6‐25.7 L assuming femoral venous temperatures of 39‐39.7°C during CE_MAX_ at an intensity equivalent to a VO_2max_ of 3.46 L min^−1^ (~285 W).^65^

#### 4.6.2 ATP production rates (APR): pmol O_2_ mg^−1^ s^−1^ to mmol ATP kg^−1^ s^−1^ & mM ATP min^−1^

Conversion from OCR (pmol O_2_ mg^−1^ s^−1^) to APR (mmol kg^−1^ s^−1^) assumed phosphate‐to‐oxygen (P/O) ratios of 2.45 for fat‐driven, 2.65 for glucose‐driven and 2.73 for glycogen‐driven respiration.^76^ Accordingly, APR conversions utilized a P/O ratio of 2.45 for FAO*_p_* and 2.72 for OXPHOS*_p_* with 2.72 reflective of 81.8% of respiration driven by skeletal muscle glycogen while the remaining 18.2% is from blood‐derived glucose.^120^

Conversion of APR from mmol kg^−1^ s^−1^ to mmol/L min^−1^ assumed a muscle density of 1.049 kg L^−1^.^80^, ^121^

#### 4.6.3 Substrate oxidation rates (SOR): mmol ATP kg^−1^ s^−1^ to kcal min^−1^ & g min^−1^

Conversion from APR (mmol kg^−1^ s^−1^) to SOR (kcal min^−1^ and then g min^−1^) assumed ΔG_ATP_ = −11.5 kcal mol^−1^,^77^, ^122^, ^123^ 4 kcal = 1 g of carbohydrate and 9 kcal = 1 g of fat.

### 4.7 Whole‐body measures of VO_2max_ to single‐leg evaluations of OCR, SOR and APR at maximal exercise

Measures of whole‐body VO_2max_ derived from standard indirect calorimetric methodologies were extrapolated to single‐leg estimates of OCR (pmol mg^−1^ s^−1^ and mL kg^−1^ min^−1^), SOR (kcal min^−1^ and g min^−1^) and APR (mmol kg^−1^ s^−1^ and mmol/L min^−1^) at maximal incremental cycling exercise with two different approaches.

The first approach initially calculated SOR (kcal min^−1^ then to g min^−1^) from whole‐body VO_2max_ (L min^−1^) assuming that 80% of oxygen consumption is accounted for by the skeletal muscle of the lower limbs,^124^ the caloric equivalent of oxygen consumption is 5.05 kcal L^−1^ O_2_ ^125^ indicating 100% CHO oxidation and 1 g of CHO is equivalent to 4 kcal. Next, SOR (kcal min^−1^) determined APR—mmol kg^−1^ s^−1^ then to mmol/L min^−1^—, which was then used (mmol kg^−1^ s^−1^) to determine OCR—pmol mg^−1^ s^−1^ to mL kg^−1^ min^−1^, as previously described.

The second approach initially determined leg VO_2max_ (mL kg^−1^ min^−1^) directly from whole‐body VO_2max_ (L min^−1^) assuming 80% of oxygen consumption is accounted for by the skeletal muscle of the lower limbs before sequential conversions were completed in the order of mL kg^−1^ min^−1^ to pmol mg^−1^ s^−1^ to APR (mmol kg^−1^ s^−1^) to SOR (kcal min^−1^), as described above. APR values of mmol/L min^−1^ and SOR values in g min^−1^ were then calculated from mmol kg^−1^ s^−1^ and kcal min^−1^ respectively.

All corresponding variables determined between approaches paired perfectly (*r* = 1.0), yet the first approach resulted in slightly yet significantly higher estimates (~3.8%). Thus, values derived from the two approaches were averaged for statistical analysis and presentation as IC‐derived measures at CE_MAX_.

### 4.8 Skeletal muscle mitochondrial temperature

Femoral venous temperatures were increased by 10.5°C to account for the thermal gradients between skeletal muscle and venous blood (~0.5°C)^126^ as well as between skeletal muscle mitochondria and skeletal muscle (~10°C).^82^, ^83^

### 4.9 Temperature correcting respiratory values

Respiratory rates derived from a standardized HRR methodology^25^, ^81^ (ie measures collected at a temperature of 37°C and high oxygen concentrations) were corrected assuming a Q_10_ of 2^26^, ^38^, ^65^:

(5) $$\text{Temperature Correction}=e^{0.0693\times (\Delta T)}$$

where Δ*T* is the difference between skeletal muscle mitochondrial temperature estimates and the temperature of the respiratory chambers during data collection (37°C).

### 4.10 Lower body skeletal muscle mass

Lower body skeletal muscle mass was estimated from anthropometric data derived using whole‐body magnetic resonance imaging across 468 non‐obese men and women from ages 18 to 88 y to determine lower body skeletal muscle mass^127^ or dual‐energy X‐ray absorptiometry across 433 healthy ambulatory individuals from ages 18 to 94 years to determine appendicular skeletal muscle mass (ASMM).^128^

Janssen *et al* (2000) J Appl Physiol (*derived from Table 1 in reference*):

(6) $$\text{Estimated Female Body Weight}\left(\text{kg}\right)=‐0.0226\times {\text{age}}^{2}+2.0454\times \text{age}+29.494$$

(7) $$\text{Estimated Female Lower Limb Mass}\left(\text{kg}\right)=‐0.0027\times {\text{age}}^{2}+0.194\times \text{age}+9.3815$$

(8) $$\text{Estimated Male Body Weight}\left(\text{kg}\right)=‐0.0215\times {\text{age}}^{2}+2.0404\times \text{age}+43.692$$

(9) $$\text{Estimated Male Lower Limb Mass}\left(\text{kg}\right)=‐0.0035\times {\text{age}}^{2}+0.2456\times \text{age}+14.546$$

Kyle *et al* (2001) Eur J Clin Nutr (*derived from Tables 1 and 2 in reference*):

(10) $$\text{Estimated Female Body Weight}\left(\text{kg}\right)=‐0.0039\times {\text{age}}^{2}+0.4442\times \text{age}+51.967$$

(11) $$\text{Estimated Female ASMM}\left(\text{kg}\right)=‐0.0004\times {\text{age}}^{2}+0.0077\times {\text{age}}^{2}+18.06$$

(12) $$\text{Estimated Male Body Weight}\left(\text{kg}\right)=‐0.0059\times {\text{age}}^{2}+0.5734\times \text{age}+65.478$$

(13) $$\text{Estimated Male Lower ASMM}\left(\text{kg}\right)=‐0.0015\times {\text{age}}^{2}+0.0926\times \text{age}+25.426$$

When appropriate, ASMM was used to determine lower body skeletal muscle mass as 78.0% and 74.6% of ASMM in women and men respectively.^129^ The method used to calculate lower body skeletal muscle mass per datum or data set was dependent on parallel estimates of body weight (kg). The approach that resulted in the closest estimate of body weight to the actual value was then used to establish respective estimates of lower body skeletal muscle mass when not directly reported in the study. Finally, lower body skeletal muscle mass estimations were adjusted based on the magnitude of difference between estimated and actual body mass by a factor of 0.153 for females and 0.168 for males.^130^

### 4.11 Glycolytic energetics at maximal exercise

Venous blood lactate concentration ([La^−^]^v^) estimates at maximal exercise were determined from a power function (*R* ^2^ = 0.8272) developed by comparing whole‐body VO_2max_, ranging from 15.1 to 79.0 mL kg^−1^ min^−1^, against [La^−^]^v^ (mmol L^−1^) using data previously collected from our research^131^ in combination with published values across other laboratories^59^, ^137^; n = 26 (Figure S6A).

(14) $${\left[{\text{La}}^{‐}\right]}^{v}=0.1052\times {\left({\text{VO}}_{2\text{max}}\right)}^{1.1604}$$

Skeletal muscle lactate concentrations ([La^−^]^sm^; mmol kg^−1^) were then determined from blood lactate estimates^138^ and converted into mmol L^−1^:

(15) $${\left[{\text{La}}^{‐}\right]}^{\text{sm}}=\left(\left({\left[{\text{La}}^{‐}\right]}^{v}‐1.2226\right)/0.5551\right)\times 1.049$$

APRs from [La^−^]^sm^ were then calculated assuming 2 ATP or 2.9 ATP produced per molecule lactate derived from glucose or glycogen respectively.^76^ Glycolytic substrates were assumed to be 18.2% blood glucose and 81.8% skeletal muscle glycogen.^120^ This glycolytic estimation was then evaluated against a separate estimate of glycolytic energy production that assumes a value of 1 mmol L^−1^ equivalent to 3 mL O_2_ kg^−1^ body mass.^99^ The two methods compared favourably (*F* = 1293, *R* ^2^ = 0.8609) with no difference in slopes (*F* = 1.40, *P* = .2370). However, the current method did result in a significantly different y‐intercept (*F* = 33.4, *P* < .0001, 95% CI =−0.006136 to 0.01357) and a small but significantly higher (*P* < .05) average estimate of glycolytic energetic contribution than the previously established method^99^ of 17.4 and 16.4% respectively, using a Wilcoxon matched pairs signed‐rank test (Figure S6B).

### 4.12 Statistical analyses

In total, 169 observations from our own research and 58 obtained from published literature outside of our laboratory were originally identified for analysis. All initial respiratory measures that qualified for analytical inclusion were first assessed to identify likely outliers using the ROUT method.^139^ Comparisons of respiratory states when controlling for aerobic fitness (mL kg^−1^ min^−1^) and flux control ratios (FAO*_p_* to OXPHOS*_p_* and OXPHOS*_p_* to ETS) were used to identify likely sample population outliers. Establishing reference values for HRR‐derived respiratory measures from permeabilized human skeletal muscle is the first aim of this study. Thus, likely outliers were removed prior to subsequent analyses and presentation unless otherwise specified. The intent is that these analyses include representative values typical for individuals when accounting for age (limited to a range 18 to 47), sex and cardiorespiratory fitness. Upon removal of statistical outliers, FAO*_p_* (n = 189), OXPHOS*_p_* (n = 211) and ETS (n = 187) were included for subsequent analysis and presentation.

A one‐way analysis of variance (ANOVA) was used to compare outcome variables across groups and methodologies. Main effects were initially determined assuming Gaussian distribution of residuals. A non‐parametric one‐way ANOVA (Kruskal‐Wallis test) was instead used and approximate *P* values reported once this assumption was violated. When significant main effects were detected, data were further analysed via Bonferroni's^140^ or Dunn's^141^ multiple‐comparison test respectively, to control for type I error. A repeated‐measures ANOVA was used to compare complementary paired ex vivo vs in vivo measures across methodologies (eg HRR‐, HRR when controlling for glycolytic influence‐ and IC‐determined VO_2max_). Our experimental design relies on matching individual values across methodologies rather than actual repeated measurements, so sphericity was assumed. Again, a Bonferroni correction was employed to control for type I error across multiple comparisons when significant main effects were detected. Simple linear regression analysis was used to describe relationships between paired ex vivo and in vivo estimates (eg one‐leg VO_2max_ derived from HRR and IC methods respectively) and complementary values of OCR relative to whole‐body VO_2max_ across methodologies (eg HRR vs a‐vO2 diff at KE_MAX_ and CE_MAX_). Regression line comparisons were conducted using a two‐tailed *F* test to calculate a *P* value first testing the null hypothesis that the slopes are all identical (the lines are parallel) and when rejecting that first null hypothesis calculating a second *P* value to test the null hypothesis that the lines are identical (comparing y‐intercepts). When comparing regression lines, we calculated the α′‐level adjusted for multiple comparisons by dividing 0.05 by the number of comparisons, k, to control for type I error^85^ (eg an α of *P* ≤ .01 is considered significant when comparing regression lines across 5 methodologies). Two‐tailed paired *t* tests or Wilcoxon matched‐pairs signed rank tests analysed variable comparisons across two groups (eg methodological comparisons in the two approaches used to estimate glycolytic contribution to total ATP production at CE_MAX_) when residuals were or were not normally distributed respectively.

All statistical evaluations were performed using a commercially available statistics program (Prism GraphPad 8.4.3; GraphPad Software, LLC; San Diego, CA, USA). An α+ of *P* ≤ .05 considered significant and data are reported as mean ± SD unless specified otherwise.

## 5 PHYSIOLOGICAL RELEVANCE

The physiological relevance of this study relates measures of human skeletal muscle respiratory control, metabolic flexibility and bioenergetics obtained via standardized high‐resolution respirometry with permeabilized skeletal muscle into a biological context that now relates to in vivo methodologies commonly utilized to assess, describe and understand human physiology. Validation of comparisons across methodologies has never before been achieved.

## CONFLICT OF INTEREST

CL is founder and CEO of Detalo Health Aps.

## Supporting information

Fig S1

Fig S2

Fig S3

Fig S4

Fig S5

Fig S6

Table S1‐S4
