# Supplementary figures and images for: Contextualizing the biological relevance of standardized high‐resolution respirometry to assess mitochondrial function in permeabilized human skeletal muscle
Source: Acta Physiol (Oxf). 2021 Mar 3;231(4):e13625. doi: 10.1111/apha.13625 (PMC8047922; doi:10.1111/apha.13625)

## ETS OCR

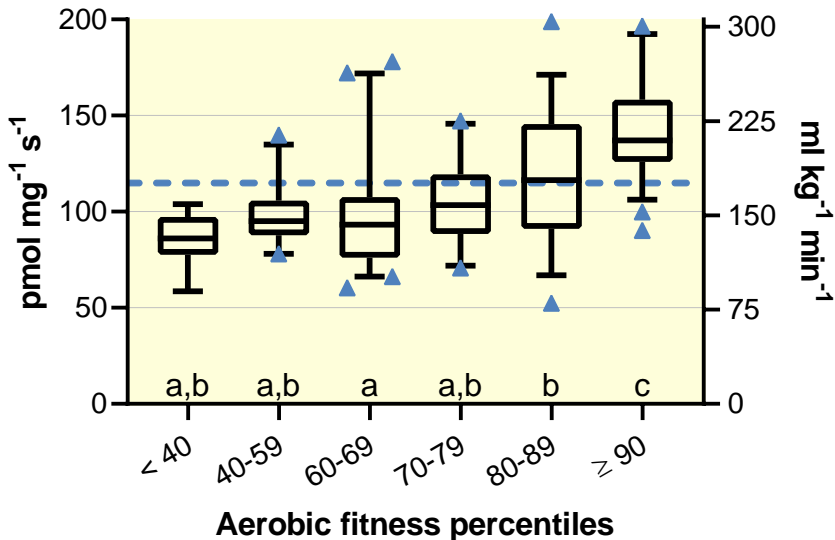

Supplement: Supplementary file 1 — Fig S1 [file APHA-231-e13625-s007.pdf]

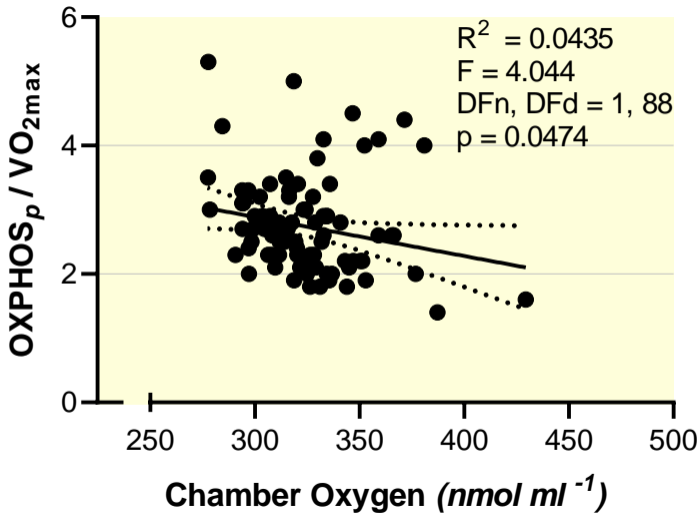

Supplement: Supplementary file 2 — Fig S2 [file APHA-231-e13625-s004.pdf]

# TEMPETS OCR

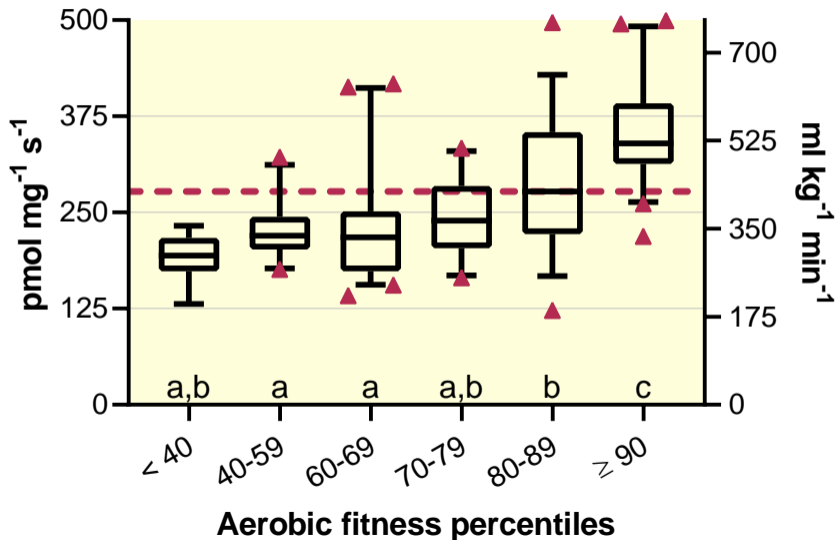

Supplement: Supplementary file 3 — Fig S3 [file APHA-231-e13625-s003.pdf]

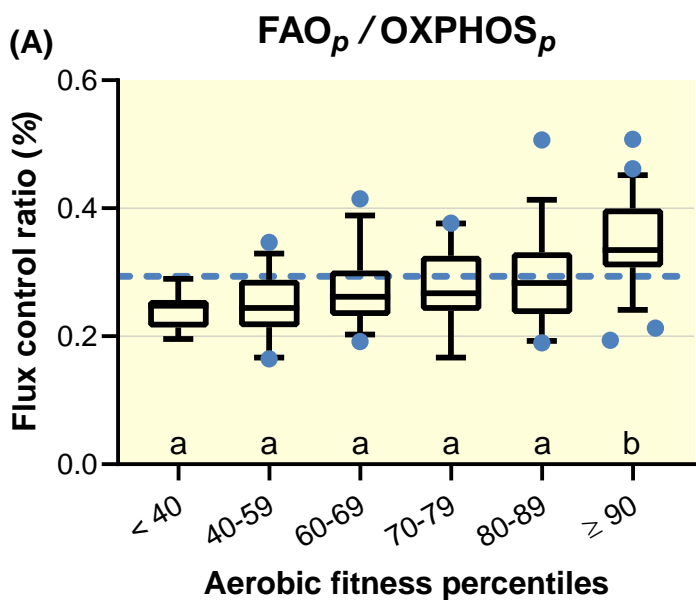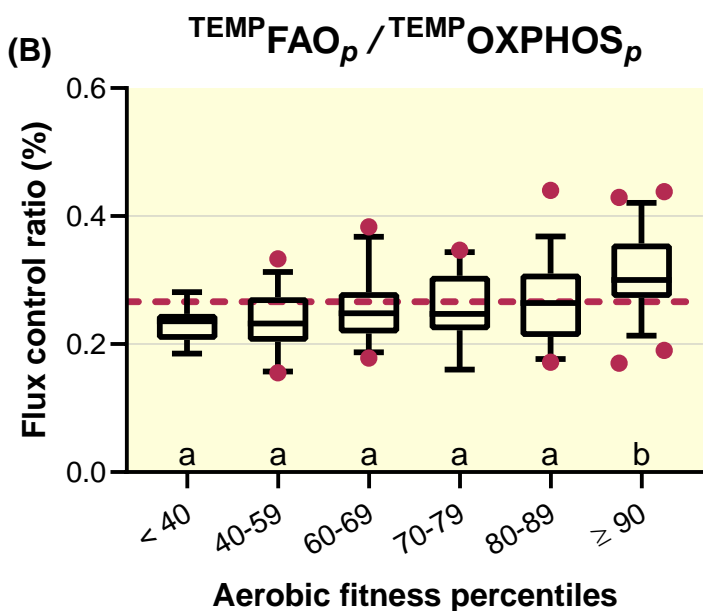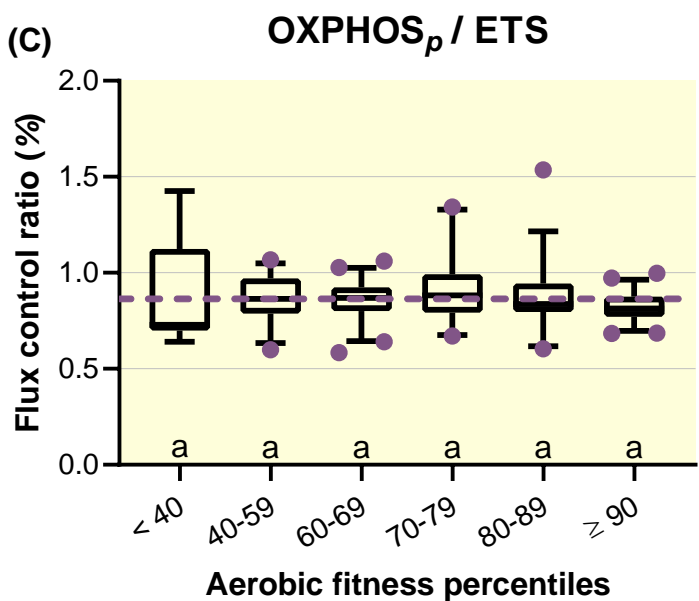

Supplement: Supplementary file 4 — Fig S4 [file APHA-231-e13625-s005.pdf]

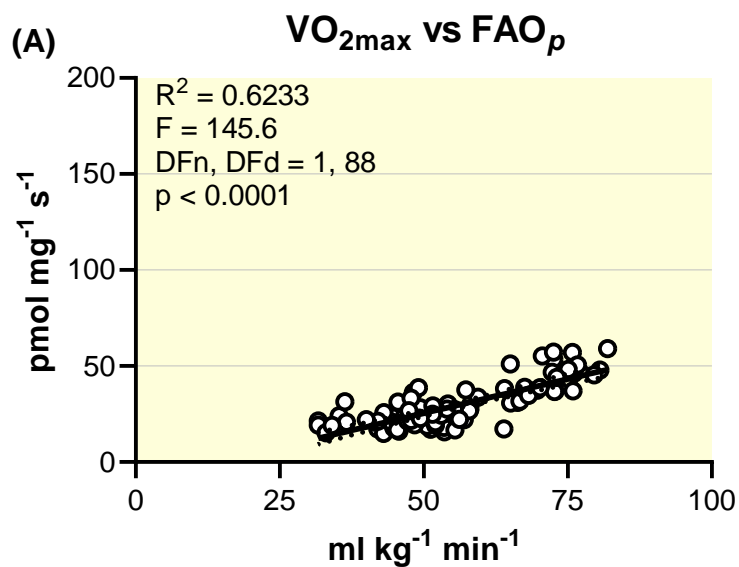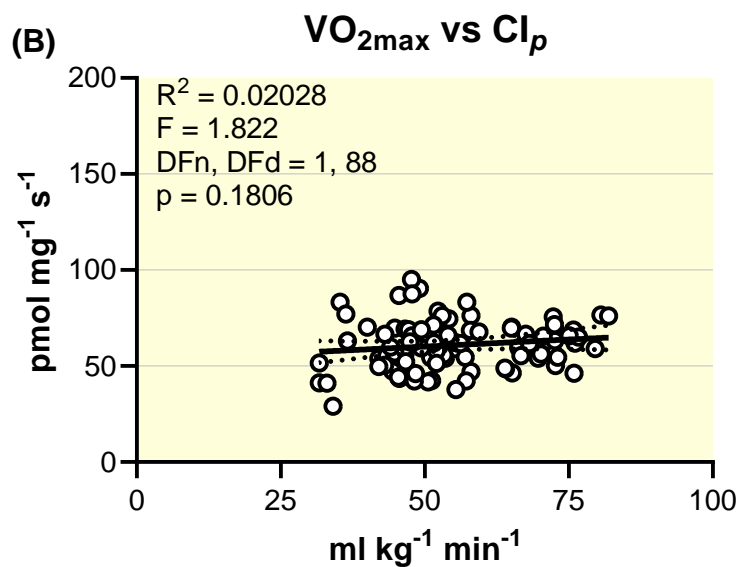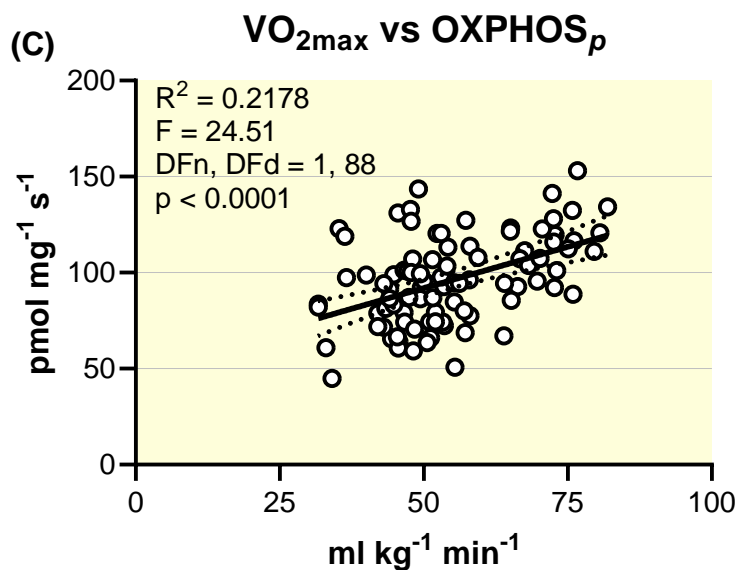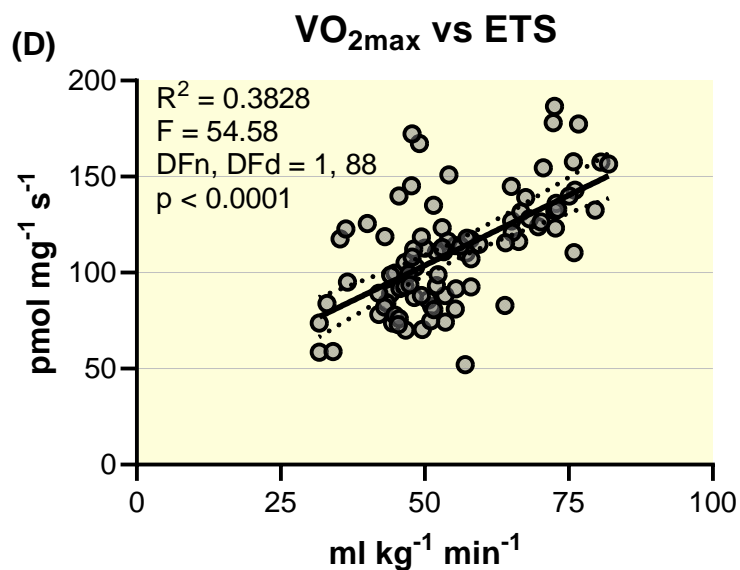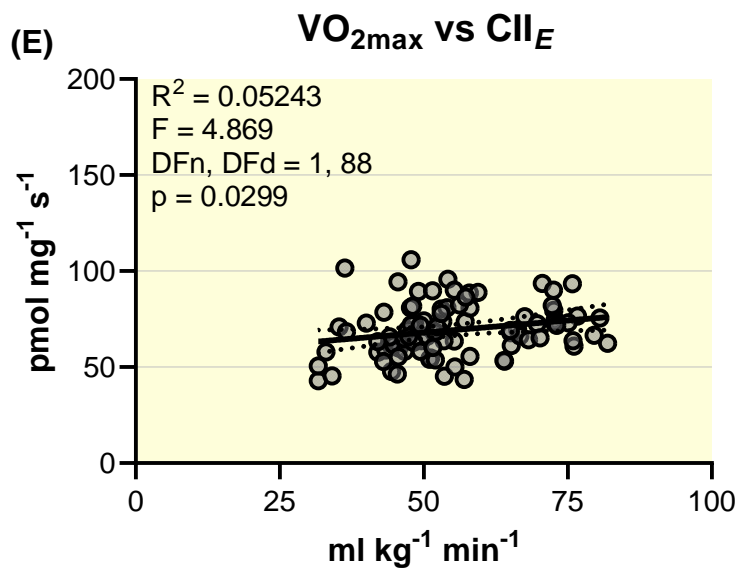

Supplement: Supplementary file 5 — Fig S5 [file APHA-231-e13625-s006.pdf]

(A)

## VO<sub>2</sub>max vs Blood Lactate

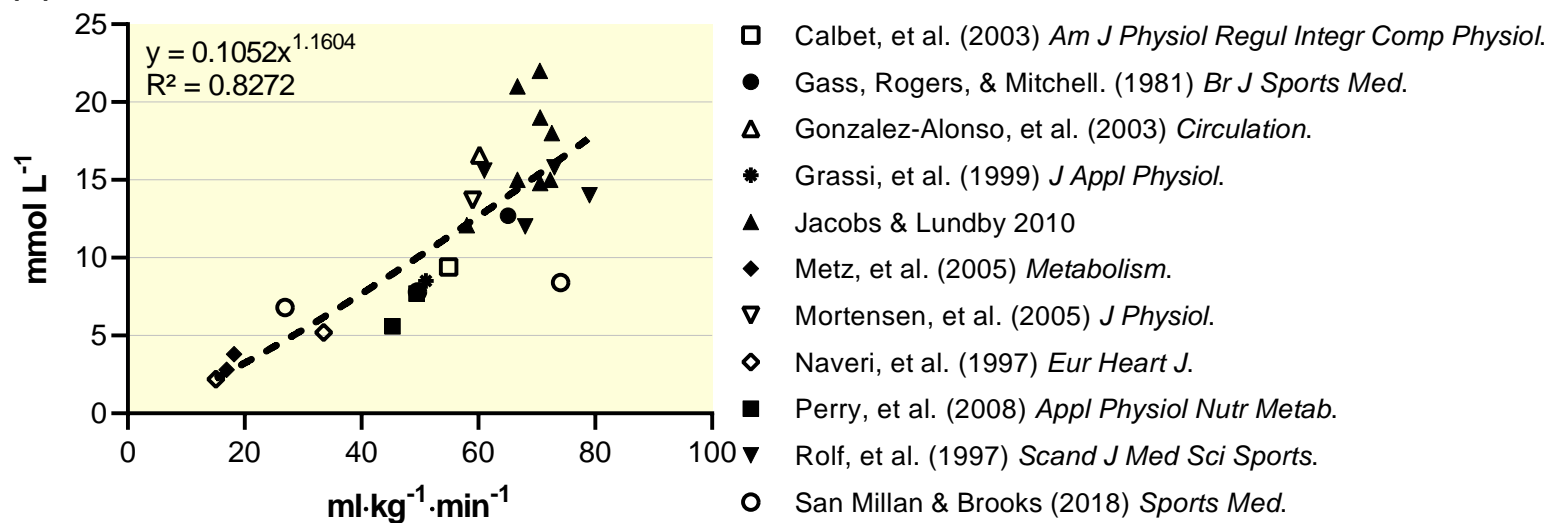

## (B) % Glycolytic ATP Provision at CE<sub>MAX</sub>

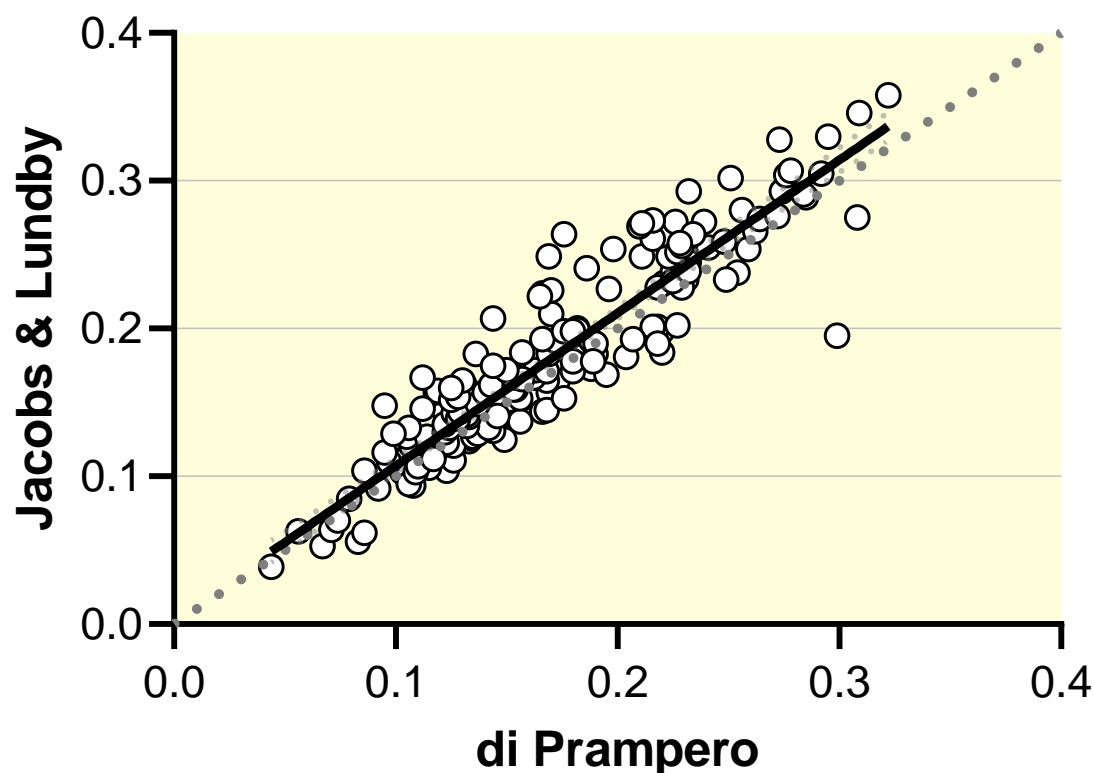

Supplement: Supplementary file 6 — Fig S6 [file APHA-231-e13625-s002.pdf]
