# Supplementary material for: Contextualizing the biological relevance of standardized high‐resolution respirometry to assess mitochondrial function in permeabilized human skeletal muscle
Source: Acta Physiol (Oxf). 2021 Mar 3;231(4):e13625. doi: 10.1111/apha.13625 (PMC8047922; doi:10.1111/apha.13625)
Supplement: Supplementary file 7 — Table S1‐S4 [file APHA-231-e13625-s001.docx]

## **Supplementary Table 1. Total group descriptive statistics for temperature controlled high-resolution respirometry-derived maximal rates of mitochondrial fatty acid oxidation (^TEMP^FAO*_p_*) from permeabilized human skeletal muscle samples.** Oxygen consumption rates (OCR); substrate oxidation rates (SOR); and ATP production rates (APR).

| **^TEMP^FAO*_p_*** | **OCR** | **OCR** | **SOR** | **SOR** | **APR** | **APR** |
| --- | --- | --- | --- | --- | --- | --- |
| **n = 189** | *pmol mg^-1^ s^-1^* | *ml kg^-1^ min^-1^* | *g min^-1^* | *kcal min^-1^* | *mmol kg^-1^ s^-1^* | *mM min^-1^* |
| **Minimum** | 23.2 | 35.4 | 0.14 | 1.27 | 0.114 | 7.1 |
| **25% Percentile** | 45.8 | 70.1 | 0.30 | 2.72 | 0.224 | 14.1 |
| **Median** | 57.6 | 88.2 | 0.39 | 3.51 | 0.282 | 17.8 |
| **75% Percentile** | 75.8 | 116.0 | 0.51 | 4.59 | 0.371 | 23.4 |
| **Maximum** | 136.5 | 208.9 | 0.90 | 8.11 | 0.669 | 42.1 |
| **Range** | 113.3 | 173.5 | 0.76 | 6.84 | 0.555 | 35.0 |
|  |  |  |  |  |  |  |
| **Mean** | 62.7 | 96.0 | 0.42 | 3.77 | 0.307 | 19.3 |
| **Std. Deviation** | 23.8 | 36.4 | 0.15 | 1.39 | 0.117 | 7.3 |
|  |  |  |  |  |  |  |
| **Lower 95% CI of mean** | 59.3 | 90.7 | 0.40 | 3.57 | 0.290 | 18.3 |
| **Upper 95% CI of mean** | 66.1 | 101.2 | 0.44 | 3.97 | 0.324 | 20.4 |
|  |  |  |  |  |  |  |
| **Coefficient of Variation** | 37.9% | 37.9% | 36.9% | 36.8% | 37.9% | 37.9% |

## **Supplementary Table 2. Total group descriptive statistics for temperature controlled high-resolution respirometry-derived maximal rates of well-coupled (P) oxidation phosphorylation (^TEMP^OXPHOS*_p_*) from permeabilized human skeletal muscle samples.** Oxygen consumption rates (OCR); substrate oxidation rates (SOR); and ATP production rates (APR).

| **^TEMP^OXPHOS*_p_*** | **OCR** | **OCR** | **SOR** | **SOR** | **APR** | **APR** |
| --- | --- | --- | --- | --- | --- | --- |
| **n = 211** | *pmol mg^-1^ s^-1^* | *ml kg^-1^ min^-1^* | *g min^-1^* | *kcal min^-1^* | *mmol kg^-1^ s^-1^* | *mM min^-1^* |
| **Minimum** | 103.4 | 158.3 | 1.48 | 5.90 | 0.561 | 35.3 |
| **25% Percentile** | 174.3 | 268.8 | 2.94 | 11.74 | 0.947 | 59.6 |
| **Median** | 221.1 | 339.4 | 3.70 | 14.81 | 1.201 | 75.6 |
| **75% Percentile** | 272.7 | 418.4 | 4.51 | 18.04 | 1.481 | 93.2 |
| **Maximum** | 435.0 | 672.8 | 7.71 | 30.83 | 2.363 | 148.7 |
| **Range** | 331.6 | 514.5 | 6.23 | 24.93 | 1.802 | 113.4 |
|  |  |  |  |  |  |  |
| **Mean** | 227.9 | 350.4 | 3.83 | 15.34 | 1.238 | 77.9 |
| **Std. Deviation** | 65.0 | 100.4 | 1.12 | 4.48 | 0.353 | 22.2 |
|  |  |  |  |  |  |  |
| **Lower 95% CI of mean** | 219.0 | 336.8 | 3.68 | 14.73 | 1.190 | 74.9 |
| **Upper 95% CI of mean** | 236.7 | 364.0 | 3.99 | 15.94 | 1.285 | 80.9 |
|  |  |  |  |  |  |  |
| **Coefficient of Variation** | 28.5% | 28.7% | 29.2% | 29.2% | 28.5% | 28.5% |

## **Supplementary Table 3. Total group descriptive statistics for temperature controlled high-resolution respirometry-derived maximal rates of non-coupled respiration representative of electron transport system capacity (^TEMP^ETS) from permeabilized human skeletal muscle samples.** Oxygen consumption rates (OCR).

| **^TEMP^ETS** | **OCR** | **OCR** |
| --- | --- | --- |
| **n = 187** | *pmol mg^-1^ s^-1^* | *ml kg^-1^ min^-1^* |
| **Minimum** | 122.3 | 187.9 |
| **25% Percentile** | 209.5 | 321.5 |
| **Median** | 267.1 | 410.3 |
| **75% Percentile** | 329.0 | 504.8 |
| **Maximum** | 494.1 | 763.3 |
| **Range** | 371.8 | 575.4 |
|  |  |  |
| **Mean** | 277.1 | 426.2 |
| **Std. Deviation** | 83.0 | 128.3 |
|  |  |  |
| **Lower 95% CI of mean** | 265.1 | 407.7 |
| **Upper 95% CI of mean** | 289.0 | 444.7 |
|  |  |  |
| **Coefficient of Variation** | 30.0% | 30.11% |

## **Supplementary Table 4. Total group descriptive statistics for excess respiratory potential corrected and temperature controlled high-resolution respirometry-derived maximal rates of well-coupled (P) oxidation phosphorylation (^ERP-TEMP^OXPHOS*_p_*) from permeabilized human skeletal muscle samples.** Oxygen consumption rates (OCR); substrate oxidation rates (SOR); and ATP production rates (APR).

| **^ERP-TEMP^OXPHOS*_p_*** | **OCR** | **OCR** | **SOR** | **SOR** | **APR** | **APR** |
| --- | --- | --- | --- | --- | --- | --- |
| **n = 211** | *pmol mg^-1^ s^-1^* | *ml kg^-1^ min^-1^* | *g min^-1^* | *kcal min^-1^* | *mmol kg^-1^ s^-1^* | *mM min^-1^* |
| **Minimum** | 51.6 | 79.0 | 0.75 | 3.01 | 0.280 | 17.6 |
| **25% Percentile** | 90.1 | 138.3 | 1.49 | 5.95 | 0.489 | 30.8 |
| **Median** | 114.8 | 176.3 | 1.92 | 7.68 | 0.624 | 39.3 |
| **75% Percentile** | 140.7 | 215.9 | 2.32 | 9.29 | 0.764 | 48.1 |
| **Maximum** | 227.6 | 352.0 | 4.03 | 16.13 | 1.236 | 77.8 |
| **Range** | 176.0 | 273.0 | 3.28 | 13.12 | 0.956 | 60.2 |
|  |  |  |  |  |  |  |
| **Mean** | 117.8 | 181.2 | 1.98 | 7.93 | 0.640 | 40.3 |
| **Std. Deviation** | 34.5 | 53.2 | 0.59 | 2.36 | 0.187 | 11.8 |
|  |  |  |  |  |  |  |
| **Lower 95% CI of mean** | 113.1 | 173.9 | 1.90 | 7.61 | 0.614 | 38.7 |
| **Upper 95% CI of mean** | 122.5 | 188.4 | 2.06 | 8.25 | 0.665 | 41.9 |
|  |  |  |  |  |  |  |
| **Coefficient of Variation** | 29.3% | 29.4% | 29.8% | 29.8% | 29.3% | 29.3% |
